# Supplementary material for: Novel Variance-Component TWAS method for studying complex human diseases with applications to Alzheimer’s dementia
Source: PLoS Genet. 2021 Apr 2;17(4):e1009482. doi: 10.1371/journal.pgen.1009482 (PMC8046351; doi:10.1371/journal.pgen.1009482)
Supplement: S1 Text — Details about PrediXcan’s and TIGAR’s approach of estimating cis-eQTL effect sizes (A, B), VC-TWAS approach with summary-level GWAS data (C), and ROS/MAP data (D). (DOCX) [file pgen.1009482.s001.docx]

**S1 Text. Details about PrediXcan’s and TIGAR’s approach of estimating cis-eQTL effect sizes, VC-TWAS approach with summary-level GWAS data, and ROS/MAP data.**

**A. PrediXcan’s approach of estimating cis-eQTL effect sizes**

PrediXcan TWAS method [1] employs Elastic-Net penalized regression method [2] to estimate cis-eQTL effect sizes $\boldsymbol{w}$ from Equation (1) in the main text. Basically, the Elastic-Net method assumes a combined LASSO ($L_{1}$) [3] and Ridge ($L_{2}$) [4] penalty and estimate $\boldsymbol{w}$ by the following equation

$$\hat{\boldsymbol{w}}=\underset{\boldsymbol{w}}{argmin} (\left\| \boldsymbol{E}_{\boldsymbol{g}}-\boldsymbol{Gw} \right\|_{2}^{2}+\lambda(\alpha{||\boldsymbol{w}||}_{1}+\frac{1}{2}(1-\alpha){||\boldsymbol{w}||}_{2}^{2})) ,$$

where $\left\| \cdot\right\|_{1}$ denotes $L_{1}$ norm, $\left\| \cdot\right\|_{2}$ denotes $L_{2}$norm. Particularly, $\alpha$ is taken as 0.5 by PrediXcan method [1] and penalty parameter $\lambda$ can be tuned by a 5-fold cross validation.

**B. TIGAR’s approach of estimating cis-eQTL effect sizes**

TIGAR^8^ provides a more flexible approach to nonparametrically estimate cis-eQTL effect sizes $\boldsymbol{w}$ from Equation (1) in the main text by a Bayesian DPR method [5]. The DPR method assumes a normal prior distribution $N\left( 0,\sigma_{w}^{2} \right)$ for cis-eQTL effect sizes and a Dirichlet process prior [6] for effect-size variance $\boldsymbol{\sigma}_{\boldsymbol{w}}^{\boldsymbol{2}}$ as follows:

$$w_{i}\sim N\left( 0,\sigma_{w}^{2} \right), \sigma_{w}^{2}\sim D, D\sim DP\left( \mathrm{IG}\left( a,b \right),\xi\right).$$

That is, the prior distribution $D$ of effect-size variance deviates from a Dirichlet Process (DP) with an Inverse Gamma (*IG*) distribution and concentration parameter $\xi$. As proposed by previous studies, variational Bayesian algorithm [7,8] is implemented to efficiently obtain posterior estimates $\hat{\boldsymbol{w}}$.

**C. VC-TWAS approach with summary-level GWAS data and P-value calculation**

VC-TWAS with summary-level GWAS data

Since summary-level GWAS data are generally generated by meta-analysis based on the following single variant (SNP) regression model:

$\boldsymbol{Y =}\boldsymbol{G}_{\boldsymbol{.j}}\beta_{j}\boldsymbol{+\varepsilon}$*,* ${{}_{i}}\sim N(0, \sigma_{\epsilon}^{2})$ .

Here, the phenotype is assumed to be adjusted for other confounding covariates with mean 0, and the genotype vectors ($\boldsymbol{G}_{\boldsymbol{.j}}$, j=1,…,m) are also assumed to be centered with mean 0. Without loss of generality, we assume GWAS summary statistics include the single variant effect size estimate $\hat{\beta}_{j}$ and corresponding standard error $\hat{\sigma_{j}}$ for the $j^{th}$ SNP, sample size $n$, and a reference LD covariance matrix $\boldsymbol{\Sigma}$ of all test SNPs.

Following the derivation provided by [9], given the marginal SNP effect size $\beta_{j}$ estimate $\hat{\beta_{j}}\boldsymbol{=}\frac{\boldsymbol{G}_{\boldsymbol{\cdot j}}^{\boldsymbol{'}}\boldsymbol{Y}}{\boldsymbol{G}_{\boldsymbol{.j}}^{\boldsymbol{'}}\boldsymbol{G}_{\boldsymbol{.j}}},$the denominator $\boldsymbol{G}_{\boldsymbol{.j}}^{\boldsymbol{'}}\boldsymbol{G}_{\boldsymbol{.j}}$ can be approximated by using the $jth$ diagonal element of the reference LD covariance matrix, $\boldsymbol{\Sigma}\approx\boldsymbol{G'G/}(n-1)$, with $\boldsymbol{G}_{\boldsymbol{.j}}^{\boldsymbol{'}}\boldsymbol{G}_{\boldsymbol{.j}}\boldsymbol{=}(n-1)\Sigma_{j,j}$. Thus, the numerator of the score statistic for the $j^{th}$ SNP as shown in the main text (Equation (6)) can be estimated by

$$\boldsymbol{G}_{\boldsymbol{\cdot j}}^{\boldsymbol{'}}\boldsymbol{Y=}\left( n-1 \right)\hat{\beta_{j}}\Sigma_{j,j} .$$

In addition, based on the estimate for the marginal SNP effect size variance, the phenotype variance $\sigma_{Y}^{2}$ can be estimated by

$\sigma_{Y}^{2}\boldsymbol{=}\frac{\boldsymbol{Y'Y}}{\left( n-1 \right)}\boldsymbol{\approx}\frac{\boldsymbol{(}\boldsymbol{G}_{\boldsymbol{.j}}^{\boldsymbol{'}}\boldsymbol{G}_{\boldsymbol{.j}}\boldsymbol{)}\hat{\sigma_{j}^{2}}(n-1)\boldsymbol{+(}\boldsymbol{G}_{\boldsymbol{.j}}^{\boldsymbol{'}}\boldsymbol{G}_{\boldsymbol{.j}}\boldsymbol{)}{\hat{\beta}_{j}}^{2}}{\left( n-1 \right)}\boldsymbol{=}\Sigma_{j,j}\hat{\sigma_{j}^{2}}(n-1)\boldsymbol{+}\Sigma_{j,j}{\hat{\beta}_{j}}^{2}\boldsymbol{.}$

Since this estimate might vary with respect to the summary GWAS data of different SNPs, we take the median of $\Sigma_{j,j}\hat{\sigma_{j}^{2}}(n-1)\boldsymbol{+}\Sigma_{j,j}{\hat{\beta}_{j}}^{2}$across all the SNPs as ${\hat{\sigma_{Y}}}^{2}$ as suggested by the previous study [9].

Then the $Q$ statistic used by VC-TWAS using only GWAS summary-level data can be approximated by

$$Q=\sum_{j=1}^{m} w_{j}^{2}\left( \frac{\boldsymbol{G}_{\boldsymbol{\cdot j}}^{\boldsymbol{'}}\boldsymbol{Y}}{{\hat{\sigma_{Y}}}^{2}} \right)^{2} .$$

P-value calculation for VC-TWAS

Under the null hypothesis, the $Q$ statistic used by VC-TWAS follows a mixture of chi-square distribution $\sum_{j=1}^{m} \lambda_{j}\chi_{j,1}^{2}$ [10,11], where ${(\lambda}_{1},\cdots,\lambda_{m})$ are nonzero eigenvalues of $\boldsymbol{\Phi}$**,**

$\boldsymbol{\Phi}\boldsymbol{=W\phi W}$**,** $\boldsymbol{\phi=}\boldsymbol{G}^{\boldsymbol{'}}\boldsymbol{PG}$***,*** $\boldsymbol{P=}\boldsymbol{V}^{\boldsymbol{-1}}\boldsymbol{-}\boldsymbol{V}^{\boldsymbol{-1}}\boldsymbol{Z}\left( \boldsymbol{Z}^{\boldsymbol{'}}\boldsymbol{V}^{\boldsymbol{-1}}\boldsymbol{Z} \right)^{\boldsymbol{-1}}\boldsymbol{Z'}\boldsymbol{V}^{\boldsymbol{-1}}$

where $\boldsymbol{G}$ is the $n\times m$ genotype matrix, $\boldsymbol{Z}$ is the matrix of covariate data,$\boldsymbol{V=}{\hat{\sigma_{Y}}}^{2}\boldsymbol{I}$ for continuous traits with identify matrix ***I***, $\boldsymbol{V=diag[}{\hat{\boldsymbol{\mu}}}_{\boldsymbol{1}}\boldsymbol{(1-}{\hat{\boldsymbol{\mu}}}_{\boldsymbol{1}}\boldsymbol{),\ldots,}{\hat{\boldsymbol{\mu}}}_{\boldsymbol{n}}\boldsymbol{(1-}{\hat{\boldsymbol{\mu}}}_{\boldsymbol{n}}\boldsymbol{)]}$ for dichotomous traits.

If phenotype $\boldsymbol{Y}$ is centered and adjusted for other covariates as assumed when using summary-level GWAS data, then $\boldsymbol{\phi}$ can be simplified and approximated by $\boldsymbol{\phi}\approx\frac{\left( n-1 \right)\boldsymbol{\Sigma}}{{\hat{\sigma_{Y}}}^{2}}$ with reference LD covariance matrix $\boldsymbol{\Sigma}$ [12].

The p-value by VC-TWAS can then be conveniently obtained from several approximation and exact methods like the Davies exact method [13], which can be done by using both individual-level and summary-level GWAS data.

**D. ROS/MAP data**

In our applications of studying Alzheimer’s dementia (AD), we used transcriptome and individual-level GWAS data generated for samples from the Religious Orders Study (ROS) and Rush Memory and Aging Project (MAP) [14-17] cohorts. ROS recruits nuns, priests, and brothers across the United States. MAP recruits participants living in private homes, subsidized housings, and retirement facilities across the greater Chicago metropolitan area. ROS/MAP data can be requested at [www.radc.rush.edu](http://www.radc.rush.edu).

Both studies employ harmonized data collection methods performed by the same staff for annual testing during life and for structured autopsy and collection of genomic data from blood and brain biospecimens. Harmonized data collection facilitates joint analyses of the studies’ data. Details of the studies are described elsewhere.

We used microarray genotype data generated for 2,093 European-decent subjects from ROS/MAP [14-17], which are further imputed to the 1000 Genome Project Phase 3 [18]. Post-mortem brain samples (gray matter of the dorsolateral prefrontal cortex) from ~30% these ROS/MAP participants with assayed genotype data are also profiled for transcriptomic data by next-generation RNA-sequencing [19], which are used as reference data to train GReX prediction models in our application studies.

Using ROS/MAP data, we conducted TWAS for clinical diagnosis of late on-site Alzheimer’s dementia (LOAD) as well as pathology indices of AD quantified with $\beta$-antibody and PHFtau specific immunostains. Quantitative pathology phenotypes $\beta$-amyloid load and PHFtau tangle density were studied. An additional phenotype of the summary measure of the burden of AD pathology (a combination of neuritic and diffuse plaques and neurofibrillary tangles based on modified Bielschowsky silver stain) [14,15,17] was also studied.

The tangle density quantifies the average PHFtau tangle density within two or more 20µm sections from eight brain regions –– hippocampus, entorhinal cortex, midfrontal cortex, inferior temporal, angular gyrus, calcarine cortex, anterior cingulate cortex, and superior frontal cortex.

The $\beta$-amyloid load quantifies the average percent area of cortex occupied by $\beta$-amyloid protein in adjacent sections from the same eight brain regions. These two are based on immunohistochemistry. The global measure of AD pathology is based on counts of neuritic and diffuse plaques and neurofibrillary tangles (15 counts) on 6µm sections stained with modified Bielschowsky [14,15,17].

**Supplementary References:**

1. Gamazon ER, Wheeler HE, Shah KP, Mozaffari SV, Aquino-Michaels K, Carroll RJ, et al. A gene-based association method for mapping traits using reference transcriptome data. Nat Genet. 2015;47(9):1091-8. Epub 2015/08/11. doi: 10.1038/ng.3367. PubMed PMID: 26258848; PubMed Central PMCID: PMCPMC4552594.

2. Zou H, Hastie T. Regularization and Variable Selection via the Elastic Net. Journal of the Royal Statistical Society Series B (Statistical Methodology). 2005;67(2):301-20.

3. Tibshirani R. Regression Shrinkage and Selection via the Lasso. Journal of the Royal Statistical Society Series B (Methodological). 1996;58(1):267-88.

4. Hoerl AE, Kennard RW. Ridge Regression: Biased Estimation for Nonorthogonal Problems. Technometrics. 2000;42(1):80-6. doi: 10.2307/1271436.

5. Zeng P, Zhou X. Non-parametric genetic prediction of complex traits with latent Dirichlet process regression models. Nat Commun. 2017;8(1):456. Epub 2017/09/08. doi: 10.1038/s41467-017-00470-2. PubMed PMID: 28878256; PubMed Central PMCID: PMCPMC5587666.

6. Muller P, Mitra R. Bayesian Nonparametric Inference - Why and How. Bayesian Anal. 2013;8(2). Epub 2013/12/26. doi: 10.1214/13-BA811. PubMed PMID: 24368932; PubMed Central PMCID: PMCPMC3870167.

7. Blei DM, Kucukelbir A, McAuliffe JD. Variational Inference: A Review for Statisticians. Journal of the American Statistical Association. 2017;112(518):859-77. doi: 10.1080/01621459.2017.1285773.

8. Carbonetto P, Stephens M. Scalable Variational Inference for Bayesian Variable Selection in Regression, and Its Accuracy in Genetic Association Studies. Bayesian Anal. 2012;7(1):73-108. doi: 10.1214/12-BA703.

9. Yang J, Ferreira T, Morris AP, Medland SE, Genetic Investigation of ATC, Replication DIG, et al. Conditional and joint multiple-SNP analysis of GWAS summary statistics identifies additional variants influencing complex traits. Nature genetics. 2012;44(4):369-75, S1-3. Epub 2012/03/20. doi: 10.1038/ng.2213. PubMed PMID: 22426310; PubMed Central PMCID: PMCPMC3593158.

10. Liu D, Lin X, Ghosh D. Semiparametric regression of multidimensional genetic pathway data: least-squares kernel machines and linear mixed models. Biometrics. 2007;63(4):1079-88. Epub 2007/12/15. doi: 10.1111/j.1541-0420.2007.00799.x. PubMed PMID: 18078480; PubMed Central PMCID: PMCPMC2665800.

11. Liu D, Ghosh D, Lin X. Estimation and testing for the effect of a genetic pathway on a disease outcome using logistic kernel machine regression via logistic mixed models. BMC Bioinformatics. 2008;9:292. Epub 2008/06/26. doi: 10.1186/1471-2105-9-292. PubMed PMID: 18577223; PubMed Central PMCID: PMCPMC2483287.

12. Lee S, Teslovich TM, Boehnke M, Lin X. General framework for meta-analysis of rare variants in sequencing association studies. American journal of human genetics. 2013;93(1):42-53. Epub 2013/06/19. doi: 10.1016/j.ajhg.2013.05.010. PubMed PMID: 23768515; PubMed Central PMCID: PMCPMC3710762.

13. Moschopoulos PG, Canada WB. The distribution function of a linear combination of chi-squares. Computers & Mathematics with Applications. 1984;10(4):383-6. doi: <https://doi.org/10.1016/0898-1221(84)90066-X>.

14. Bennett DA, Schneider JA, Arvanitakis Z, Wilson RS. Overview and findings from the religious orders study. Curr Alzheimer Res. 2012;9(6):628-45. Epub 2012/04/05. PubMed PMID: 22471860; PubMed Central PMCID: PMCPMC3409291.

15. Bennett DA, Schneider JA, Buchman AS, Barnes LL, Boyle PA, Wilson RS. Overview and findings from the rush Memory and Aging Project. Curr Alzheimer Res. 2012;9(6):646-63. Epub 2012/04/05. doi: 10.2174/156720512801322663. PubMed PMID: 22471867; PubMed Central PMCID: PMCPMC3439198.

16. Ng B, White CC, Klein HU, Sieberts SK, McCabe C, Patrick E, et al. An xQTL map integrates the genetic architecture of the human brain's transcriptome and epigenome. Nat Neurosci. 2017;20(10):1418-26. Epub 2017/09/05. doi: 10.1038/nn.4632. PubMed PMID: 28869584; PubMed Central PMCID: PMCPMC5785926.

17. Bennett DA, Buchman AS, Boyle PA, Barnes LL, Wilson RS, Schneider JA. Religious Orders Study and Rush Memory and Aging Project. J Alzheimers Dis. 2018;64(s1):S161-S89. Epub 2018/06/06. doi: 10.3233/JAD-179939. PubMed PMID: 29865057; PubMed Central PMCID: PMCPMC6380522.

18. Buchanan CC, Torstenson ES, Bush WS, Ritchie MD. A comparison of cataloged variation between International HapMap Consortium and 1000 Genomes Project data. J Am Med Inform Assoc. 2012;19(2):289-94. Epub 2012/02/10. doi: 10.1136/amiajnl-2011-000652. PubMed PMID: 22319179; PubMed Central PMCID: PMCPMC3277631.

19. De Jager PL, Srivastava G, Lunnon K, Burgess J, Schalkwyk LC, Yu L, et al. Alzheimer's disease: early alterations in brain DNA methylation at ANK1, BIN1, RHBDF2 and other loci. Nat Neurosci. 2014;17(9):1156-63. Epub 2014/08/19. doi: 10.1038/nn.3786. PubMed PMID: 25129075; PubMed Central PMCID: PMCPMC4292795.
