# Supplementary figures and images for: Novel Variance-Component TWAS method for studying complex human diseases with applications to Alzheimer’s dementia
Source: PLoS Genet. 2021 Apr 2;17(4):e1009482. doi: 10.1371/journal.pgen.1009482 (PMC8046351; doi:10.1371/journal.pgen.1009482)

**(A)**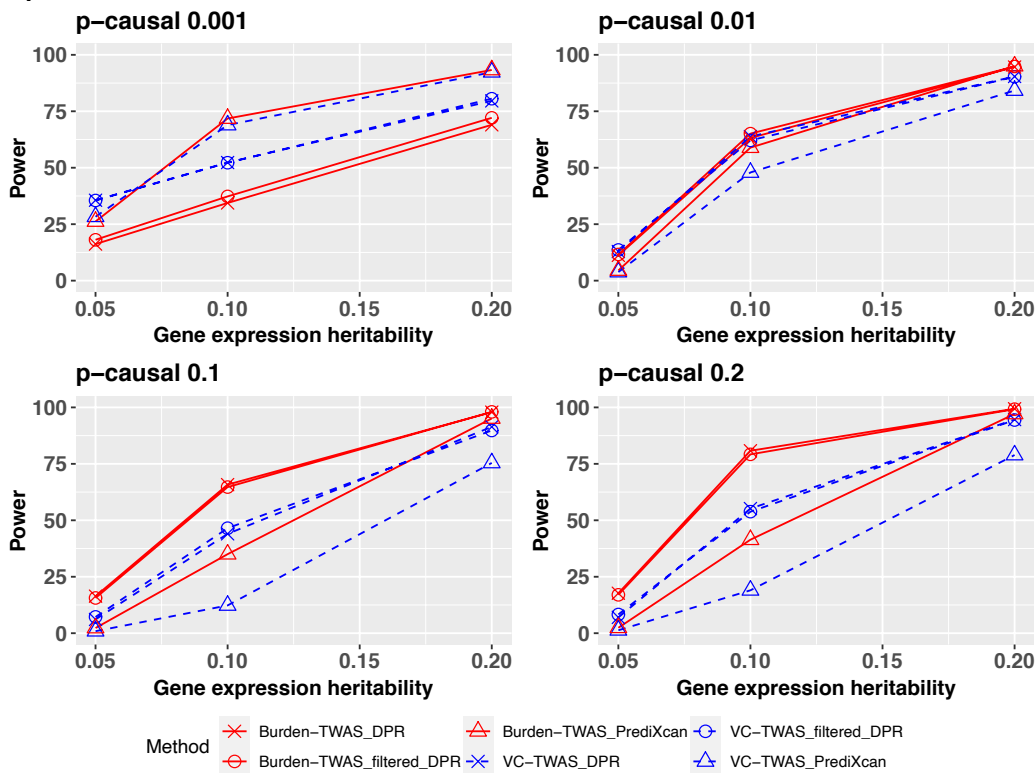**(B)**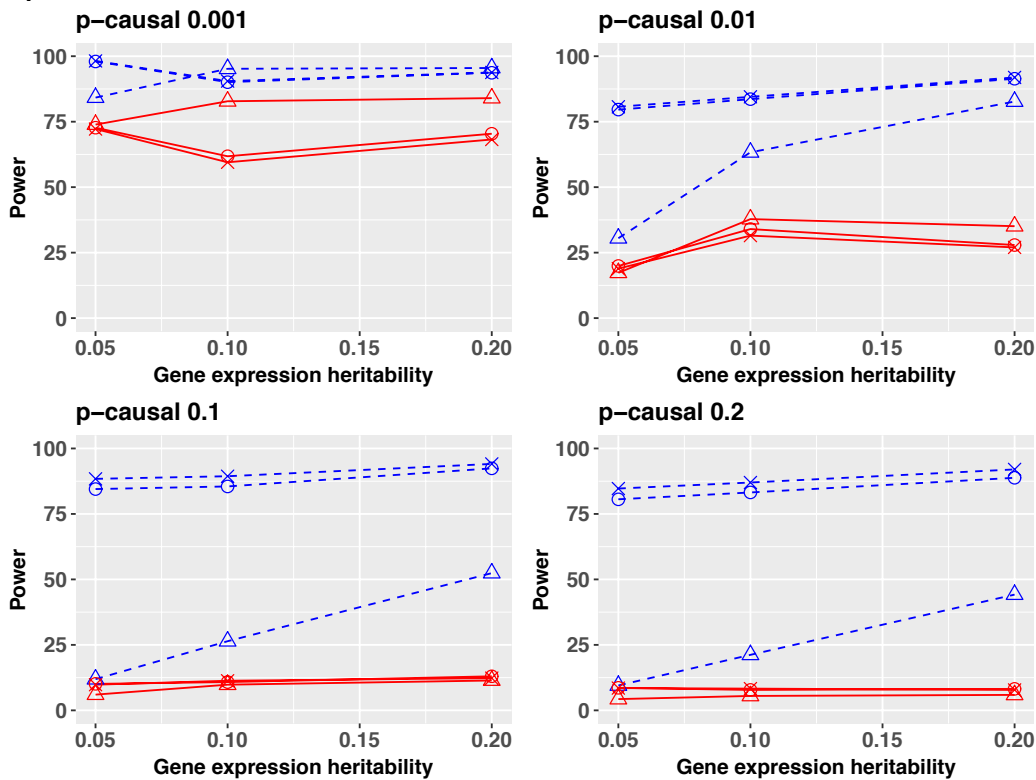

Supplement: S1 Fig — TWAS power comparison for VC-TWAS and Burden-TWAS with phenotypes simulated from Model I (A) and Model II (B). Various types of SNP weights were considered, including those derived from PrediXcan method, DPR method, and filtered DPR weights. In Model I, the combinations of causal probability and phenotype heritability are (pcausal,hp2)=((0.001,0.2),(0.01,0.3),(0.1,0.4),(0.2,0.5)). In Model II, the combinations of causal probability and phenotype heritability are (pcausal,hp2)=((0.001,0.1),(0.01,0.1),(0.1,0.15),(0.2,0.15)). (PDF) [file pgen.1009482.s002.pdf]

**A) Model I**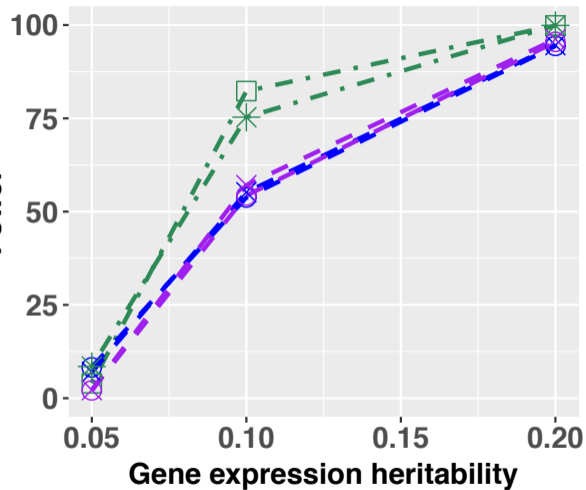**B) Model II**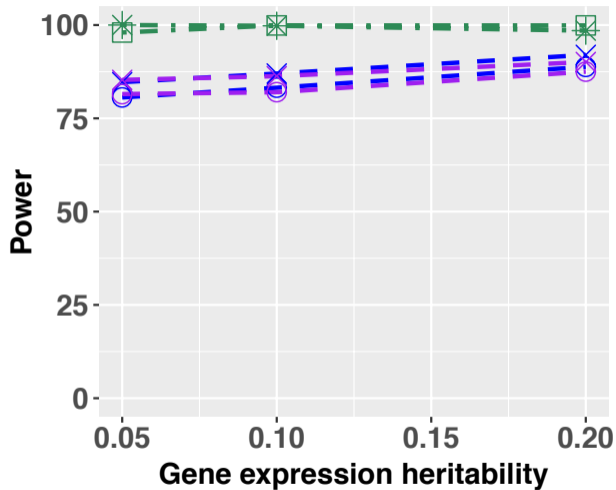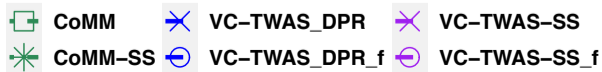

Supplement: S2 Fig — TWAS power comparison for VC-TWAS and CoMM with phenotypes simulated from Model I (A) and Model II (B) using individual-level and summary-level data under the scenarios with pcausal = 0.2. (PDF) [file pgen.1009482.s003.pdf]

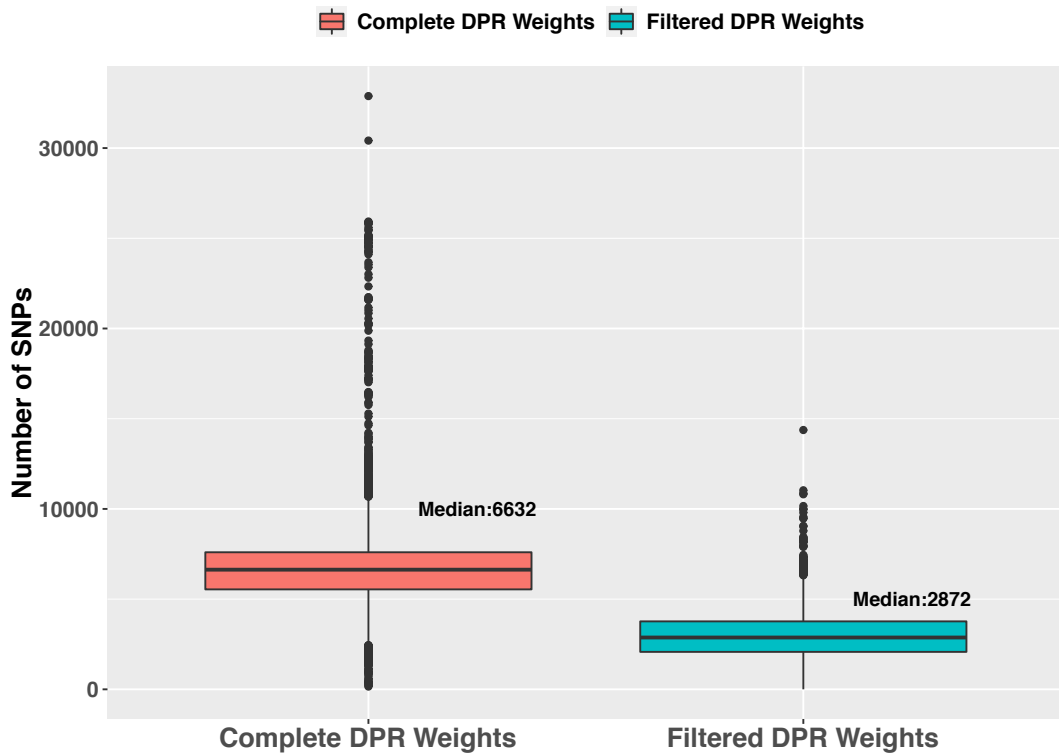

Supplement: S3 Fig — (PDF) [file pgen.1009482.s004.pdf]

A)

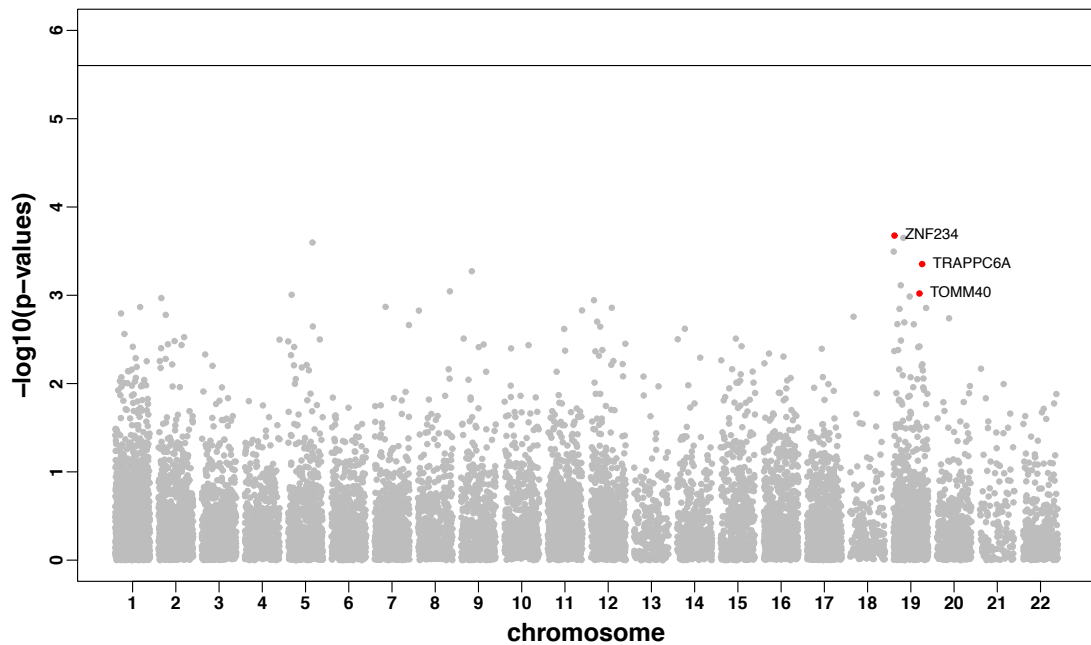

B)

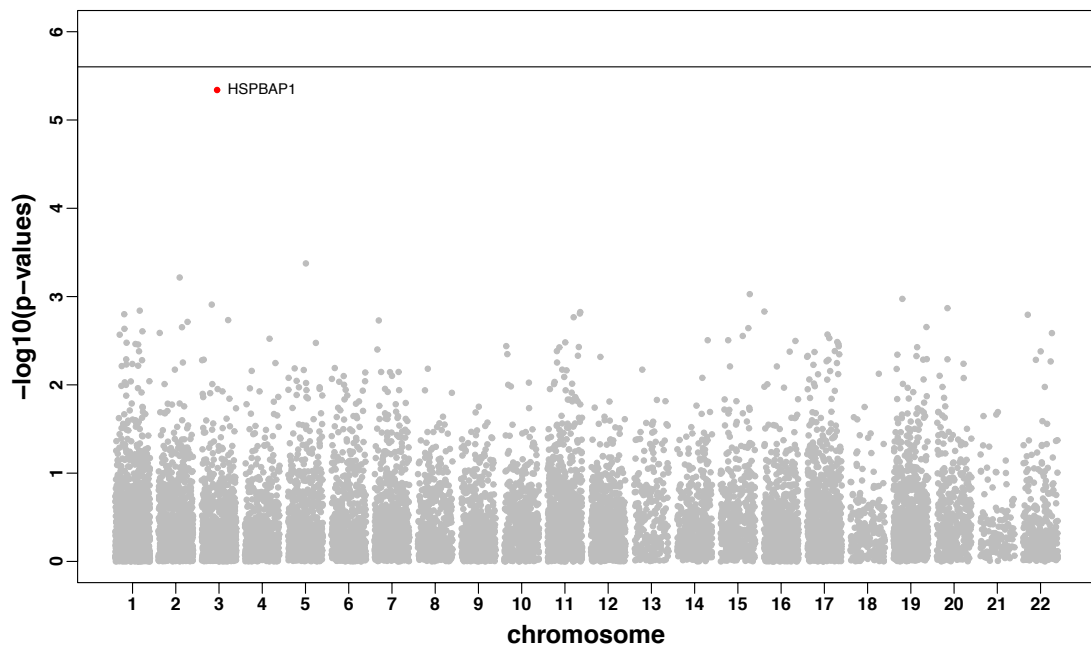

Supplement: S5 Fig — Manhattan plots of VC-TWAS results with filtered DPR weights for studying quantitative AD pathology of β-Amyloid (A) and tangles (B). Genes with FDA < 0.05 by meta VC-TWAS for studying AD clinical diagnosis are colored in red in (A) and top significant gene for studying tangles phenotype with FDR = 0.058 is colored in red in (B). (PDF) [file pgen.1009482.s006.pdf]

A)

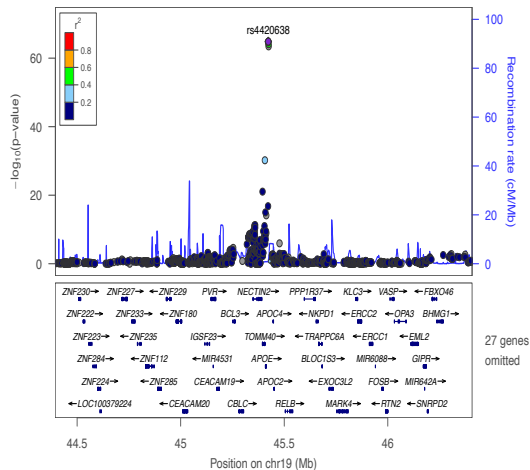

C)

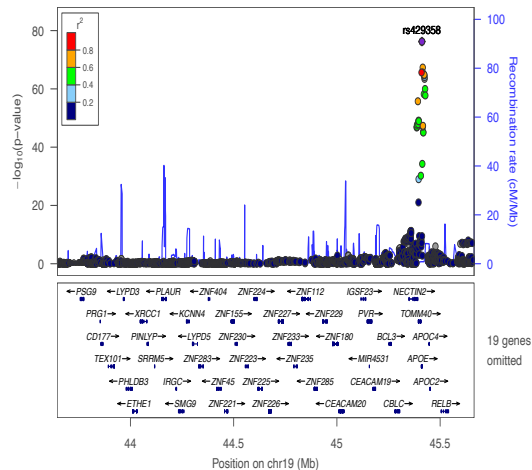

B)

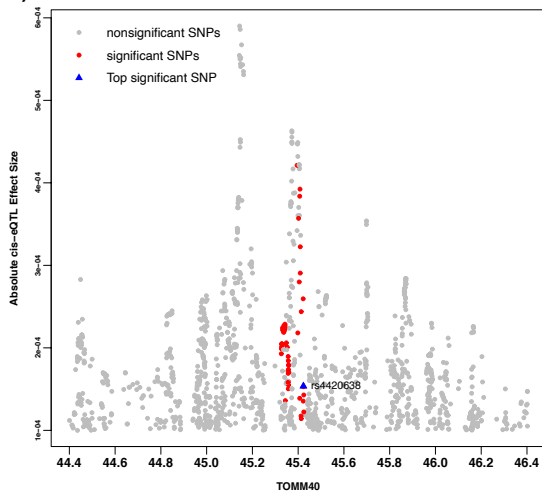

D)

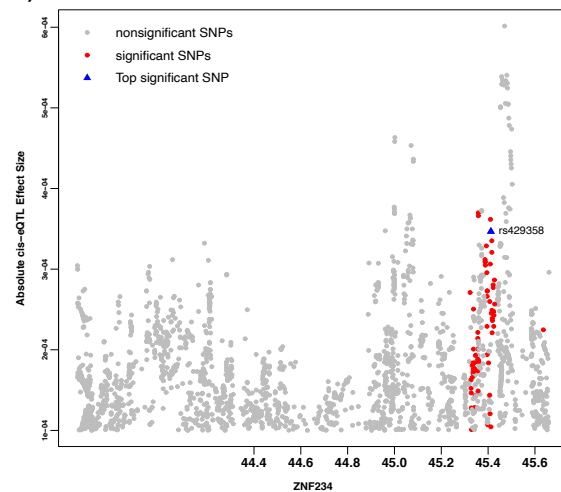

Supplement: S7 Fig — Locus zoom plots of GWAS results and the magnitude (i.e., absolute value) of cis-eQTL effect size estimates by DPR method for SNPs that were considered by VC-TWAS of genes TOMM40 (A, B) and ZNF2334 (C, D). Filtered test SNPs with the cis-eQTL effect size magnitude > 10−4 were plotted here. SNPs with GWAS p-value <5×10^−8^ were colored in red in (B,D), top significant SNPs by GWAS in (A,C) were shown as the blue triangle in (B,D). (PDF) [file pgen.1009482.s008.pdf]

**A)**

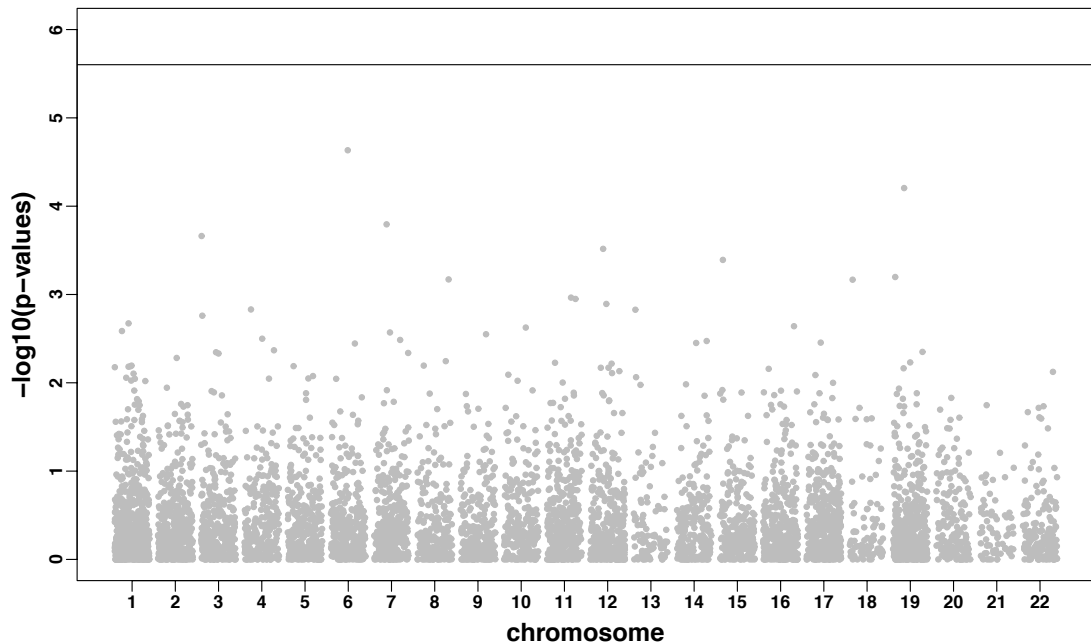

**B)**

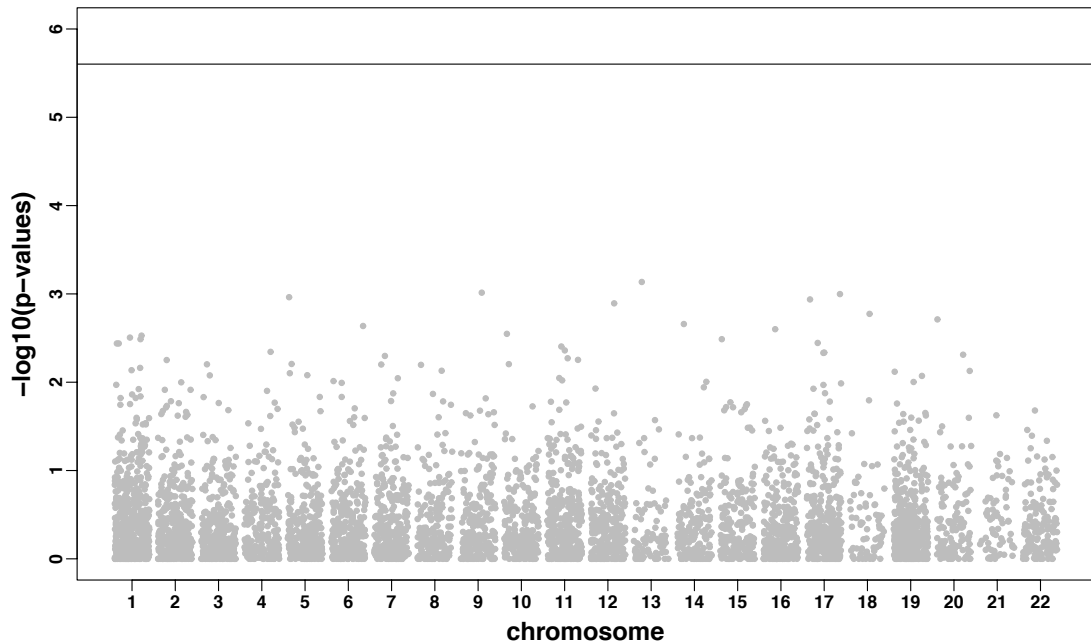

Supplement: S8 Fig — Manhattan plots of VC-TWAS results with PrediXcan weights for studying AD clinical diagnosis (A) and global AD pathology (B). (PDF) [file pgen.1009482.s009.pdf]

A)

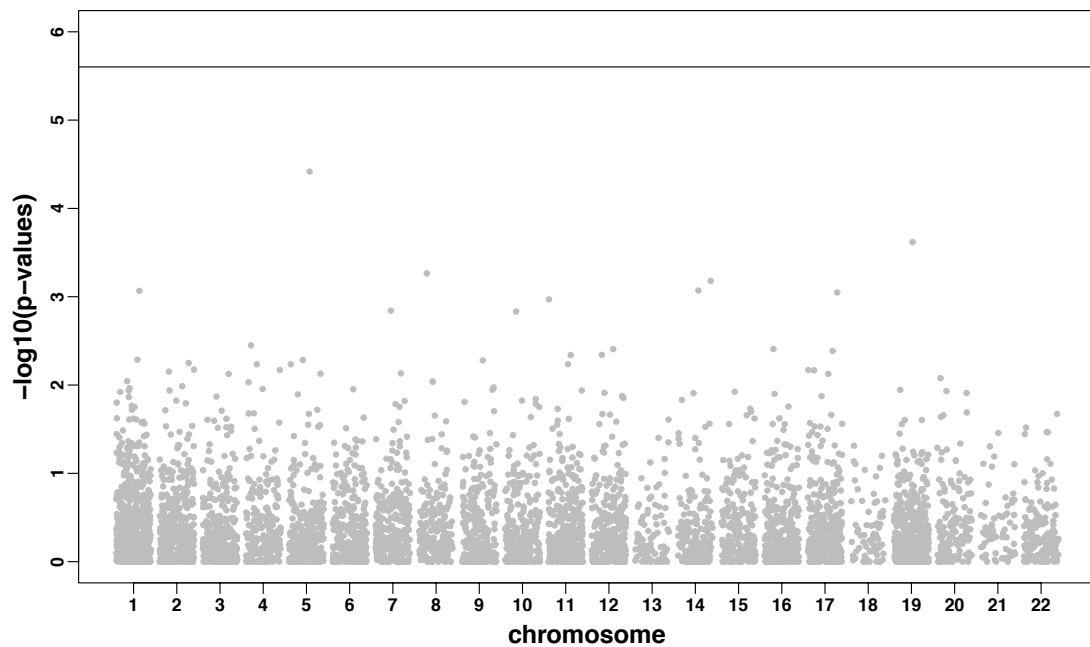

B)

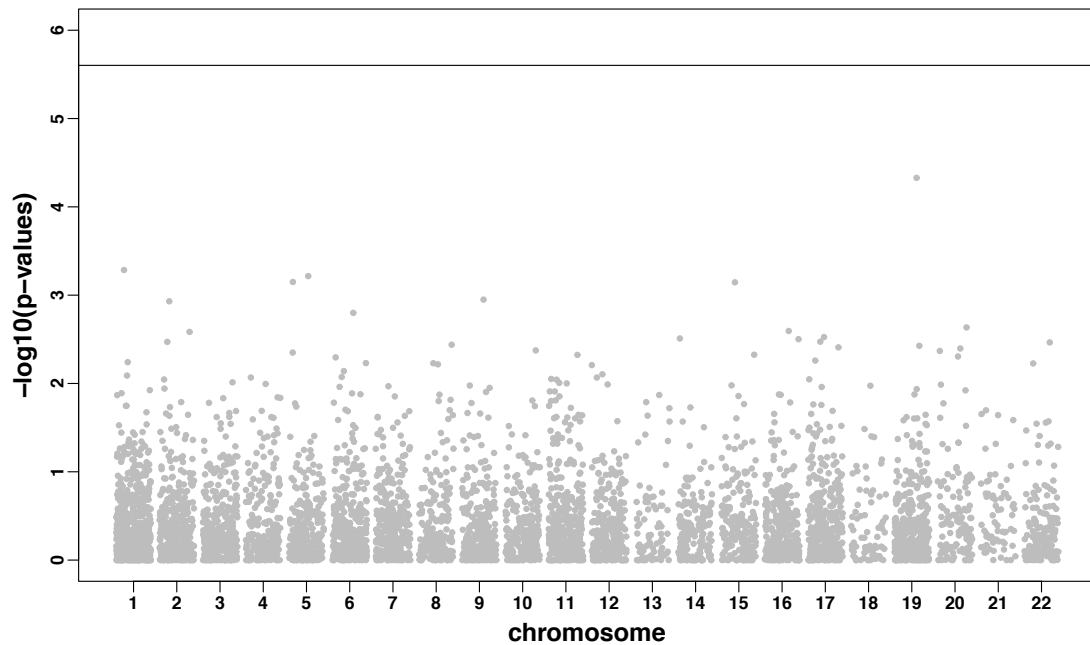

Supplement: S9 Fig — Manhattan plots of VC-TWAS results with PrediXcan weights for studying quantitative AD pathology of β-Amyloid (A) and tangles (B). (PDF) [file pgen.1009482.s010.pdf]

**A)**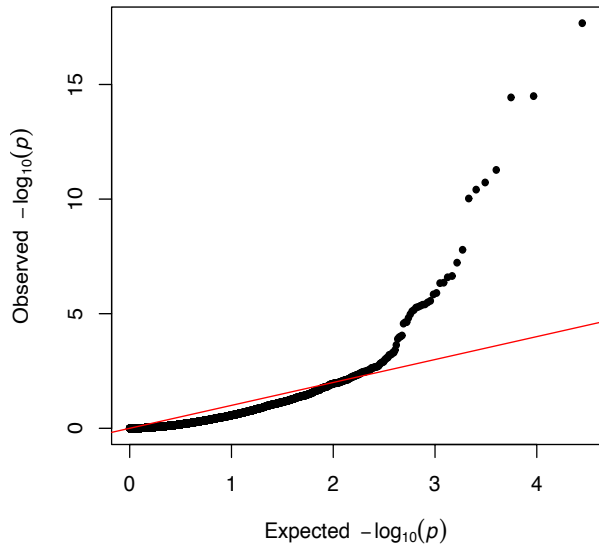**B)**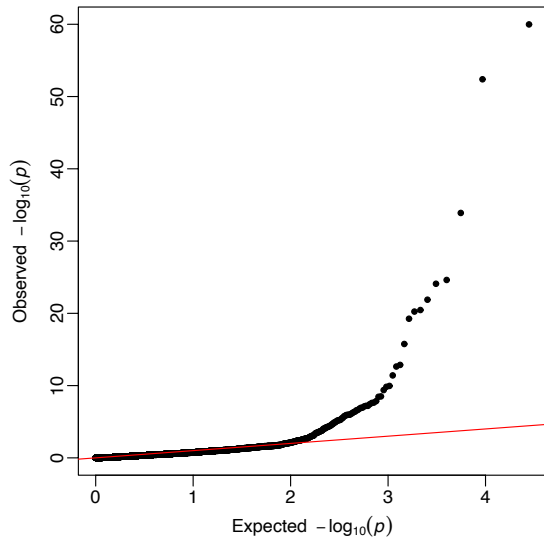

Supplement: S11 Fig — (PDF) [file pgen.1009482.s012.pdf]
