# Supplementary material for: Novel Variance-Component TWAS method for studying complex human diseases with applications to Alzheimer’s dementia
Source: PLoS Genet. 2021 Apr 2;17(4):e1009482. doi: 10.1371/journal.pgen.1009482 (PMC8046351; doi:10.1371/journal.pgen.1009482)
Supplement: S4 Fig — (PDF) [file pgen.1009482.s005.pdf]

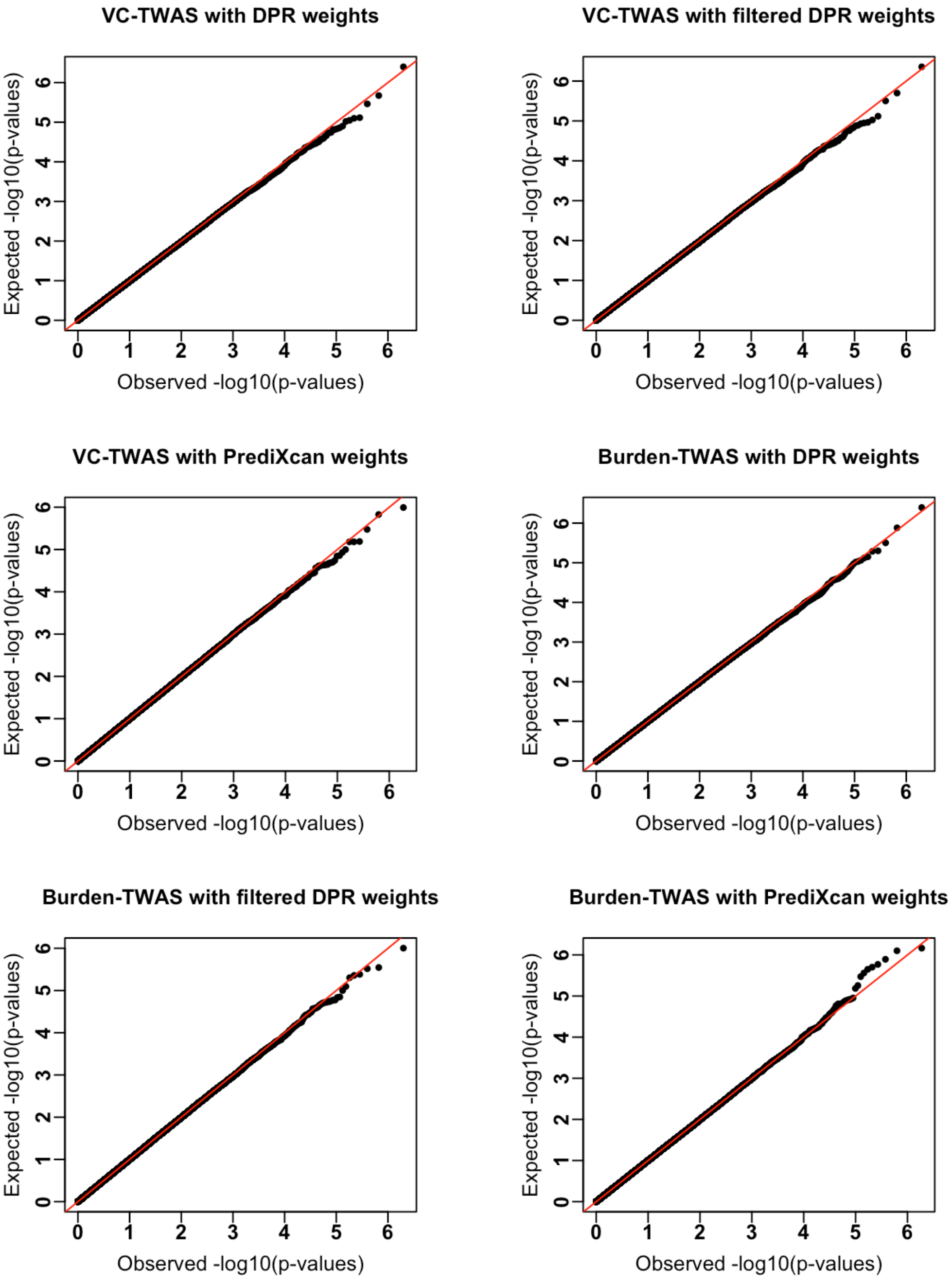
VC-TWAS with DPR weights
VC-TWAS with filtered DPR weights
VC-TWAS with PrediXcan weights
Burden-TWAS with DPR weights
Burden-TWAS with filtered DPR weights
Burden-TWAS with PrediXcan weights
Expected -log10(p-values)
Observed -log10(p-values)
0
1
2
3
4
5
6
