# Supplementary material for: Novel Variance-Component TWAS method for studying complex human diseases with applications to Alzheimer’s dementia
Source: PLoS Genet. 2021 Apr 2;17(4):e1009482. doi: 10.1371/journal.pgen.1009482 (PMC8046351; doi:10.1371/journal.pgen.1009482)
Supplement: S6 Fig — (PDF) [file pgen.1009482.s007.pdf]

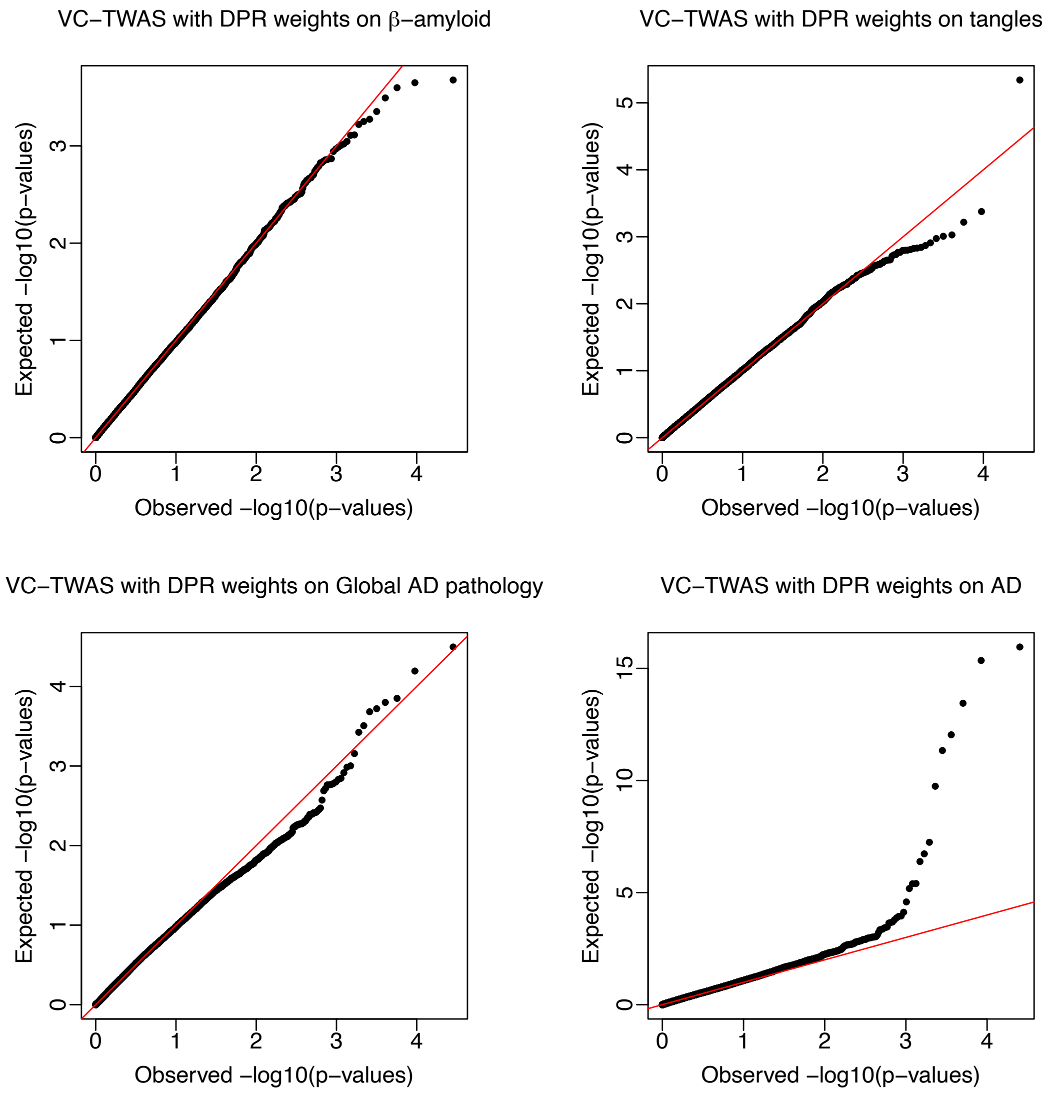
VC−TWAS with DPR weights on β−amyloid
VC−TWAS with DPR weights on tangles
VC−TWAS with DPR weights on Global AD pathology
VC−TWAS with DPR weights on AD
Expected −log10(p−values)
Observed −log10(p−values)
0
1
2
3
4
5
10
15
