# Supplementary material for: Novel Variance-Component TWAS method for studying complex human diseases with applications to Alzheimer’s dementia
Source: PLoS Genet. 2021 Apr 2;17(4):e1009482. doi: 10.1371/journal.pgen.1009482 (PMC8046351; doi:10.1371/journal.pgen.1009482)
Supplement: S10 Fig — (PDF) [file pgen.1009482.s011.pdf]

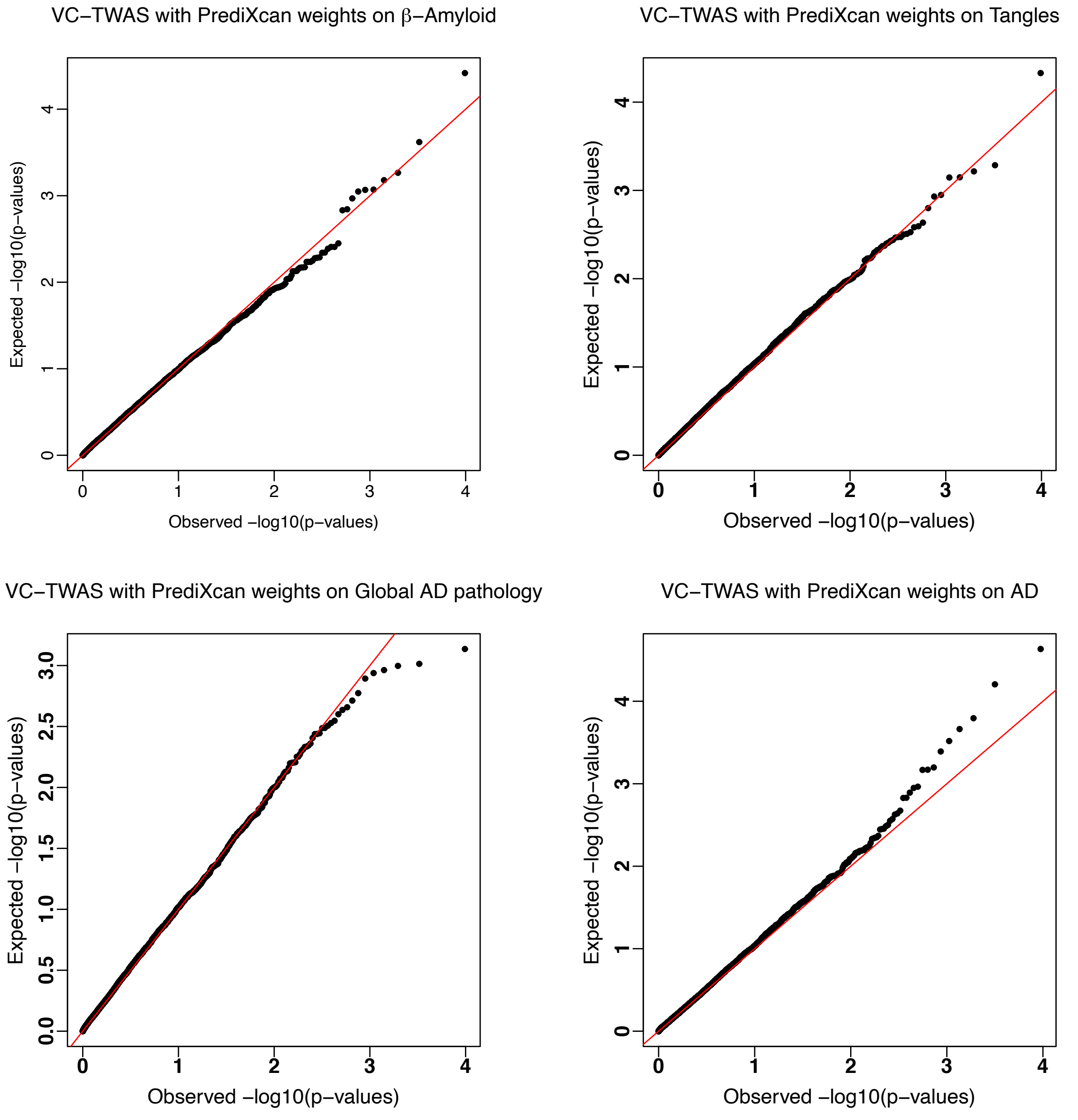

VC−TWAS with PrediXcan weights on β−Amyloid
Expected −log10(p−values)
Observed −log10(p−values)
VC−TWAS with PrediXcan weights on Tangles
Expected −log10(p−values)
Observed −log10(p−values)
VC−TWAS with PrediXcan weights on Global AD pathology
Expected −log10(p−values)
Observed −log10(p−values)
VC−TWAS with PrediXcan weights on AD
Expected −log10(p−values)
Observed −log10(p−values)
