# Supplementary material for: Novel Variance-Component TWAS method for studying complex human diseases with applications to Alzheimer’s dementia
Source: PLoS Genet. 2021 Apr 2;17(4):e1009482. doi: 10.1371/journal.pgen.1009482 (PMC8046351; doi:10.1371/journal.pgen.1009482)
Supplement: S1 Table — (DOCX) [file pgen.1009482.s013.docx]

**S1 Table.** Average computation time per gene (in the unit of second) by all methods with individual and summary-level GWAS data by a single thread with 4 cores (32GB memory) from a 2.10GHZ CPU 16-core Intel Xeon computation node, for example genes that have test SNP numbers in respective range.

| **SNP number** | **Burden-TWAS** | **Burden-TWAS-SS** | **VC-TWAS** | **VC-TWAS-SS** | **CoMM** | **CoMM-SS** | **PMR** | **PMR-SS** |
| --- | --- | --- | --- | --- | --- | --- | --- | --- |
| 100-300 | 0.11 | 0.0017 | 0.23 | 0.074 | 0.8689 | 0.64 | 2.55 | 0.40 |
| 300-1000 | 0.25 | 0.0016 | 0.28 | 0.15 | 73.62 | 2.11 | 40.09 | 13.26 |
| 1000-2000 | 0.28 | 0.0039 | 4.73 | 1.42 | 2333.62 | 81.93 | 1619.16 | 506 |
| 2000-5000 | 0.26 | 0.0124 | 20.09 | 3.71 | 38037.01 | 373.16 | 11681.42 | 5666.40 |
| >5000 | 0.25 | 0.0478 | 138.93 | 20.64 | 83349.44 | 5476.43 | 153506.36 | 119568.27 |
