# Supplementary material for: Novel Variance-Component TWAS method for studying complex human diseases with applications to Alzheimer’s dementia
Source: PLoS Genet. 2021 Apr 2;17(4):e1009482. doi: 10.1371/journal.pgen.1009482 (PMC8046351; doi:10.1371/journal.pgen.1009482)
Supplement: S2 Table — AD risk genes identified by previous GWAS are shaded in grey. (DOCX) [file pgen.1009482.s014.docx]

**S2 Table.** Genes with VC-TWAS p-value <0.0013 with respect to at least one AD pathology phenotype and FDR <0.05 by meta VC-TWAS of AD clinical diagnosis. AD risk genes identified by previous GWAS are shaded in grey.

| **Gene** | **CHROM** | $\boldsymbol{\beta}$**-Amyloid** | **Tangles** | **Global AD pathology** |
| --- | --- | --- | --- | --- |
| *ZNF234* | 19 | $2.10\times{10}^{-4}$^*^ | $1.06\times{10}^{-3}$^*^ | $6.39\times{10}^{-5}$^*^ |
| *CLASRP* | 19 | $1.39\times{10}^{-3}$ | $8.69\times{10}^{-3}$ | $3.76\times{10}^{-4}$^*^ |
| *TRAPPC6A* | 19 | $4.44\times{10}^{-4}$^*^ | $3.74\times{10}^{-3}$ | $1.91\times{10}^{-4}$^*^ |
| *TOMM40* | 19 | $9.55\times{10}^{-4}$^*^ | $6.95\times{10}^{-2}$ | $2.08\times{10}^{-4}$^*^ |
| *CEACAM19* | 19 | $1.03\times{10}^{-3}$^*^ | $1.21\times{10}^{-2}$ | $3.19\times{10}^{-5}$^*^ |
