# Supplementary material for: Novel Variance-Component TWAS method for studying complex human diseases with applications to Alzheimer’s dementia
Source: PLoS Genet. 2021 Apr 2;17(4):e1009482. doi: 10.1371/journal.pgen.1009482 (PMC8046351; doi:10.1371/journal.pgen.1009482)
Supplement: S3 Table — Significant genes were identified with FDR < 0.05. AD risk genes identified by previous GWAS are shaded in grey. (DOCX) [file pgen.1009482.s015.docx]

**S3 Table.** Significant genes identified by VC-TWAS using IGAP summary statistics with filtered cis-eQTL DPR weights. Significant genes were identified with FDR < 0.05. AD risk genes identified by previous GWAS are shaded in grey.

| **Gene name** | **CHROM** | **Start** | **End** | **P-value** | **FDR** |
| --- | --- | --- | --- | --- | --- |
| *CUTA* | 6 | 33,384,218 | 33,386,094 | $1.95\times{10}^{-5}$ | $5.96\times{10}^{-3}$ |
| *CLU* | 8 | 27,454,433 | 27,472,548 | $2.01\times{10}^{-5}$ | $6.13\times{10}^{-3}$ |
| *OSBP* | 11 | 59,341,870 | 59,383,617 | $8.67\times{10}^{-6}$ | $2.84\times{10}^{-3}$ |
| *STX3* | 11 | 59,480,928 | 59,573,354 | $2.82\times{10}^{-5}$ | $8.09\times{10}^{-3}$ |
| *PRPF19* | 11 | 60,658,201 | 60,674,060 | $2.93\times{10}^{-6}$ | $1.00\times{10}^{-3}$ |
| *TMEM109* | 11 | 60,681,345 | 60,690,915 | $3.66\times{10}^{-5}$ | $1.03\times{10}^{-2}$ |
| *TMEM132A* | 11 | 60,691,934 | 60,704,631 | $3.56\times{10}^{-6}$ | $1.19\times{10}^{-3}$ |
| *ME3* | 11 | 86,152,149 | 86,383,678 | $1.52\times{10}^{-5}$ | $4.76\times{10}^{-3}$ |
| *ZNF221* | 19 | 44,455,379 | 44,471,752 | $5.73\times{10}^{-5}$ | $1.55\times{10}^{-2}$ |
| *ZNF230* | 19 | 44,507,076 | 44,518,072 | $8.75\times{10}^{-20}$ | $6.48\times{10}^{-17}$ |
| *ZNF222* | 19 | 44,529,493 | 44,537,260 | $1.58\times{10}^{-11}$ | $6.16\times{10}^{-9}$ |
| *ZNF284^b^* | 19 | 44,576,296 | 44,591,623 | $6.39\times{10}^{-11}$ | $2.43\times{10}^{-8}$ |
| *ZNF225 ^a^* | 19 | 44,617,547 | 44,637,255 | $6.77\times{10}^{-8}$ | $2.51\times{10}^{-5}$ |
| *ZNF234 ^a, b^* | 19 | 44,645,709 | 44,664,462 | $3.38\times{10}^{-57}$ | $1.19\times{10}^{-53}$ |
| *ZNF226* | 19 | 44,669,214 | 44,681,836 | $7.69\times{10}^{-7}$ | $2.70\times{10}^{-4}$ |
| *ZNF227 ^b^* | 19 | 44,716,690 | 44,741,420 | $4.83\times{10}^{-18}$ | $3.24\times{10}^{-15}$ |
| *ZNF233* | 19 | 44,754,317 | 44,815,771 | $2.78\times{10}^{-5}$ | $8.09\times{10}^{-3}$ |
| *ZFP112 ^b^* | 19 | 44,830,705 | 44,905,774 | $7.16\times{10}^{-13}$ | $3.05\times{10}^{-10}$ |
| *PVR ^b^* | 19 | 45,147,097 | 45,169,429 | $3.28\times{10}^{-14}$ | $1.65\times{10}^{-11}$ |
| *CEACAM19 ^a,b^* | 19 | 45,174,723 | 45,187,631 | $7.27\times{10}^{-27}$ | $7.30\times{10}^{-24}$ |
| *BCL3 ^b^* | 19 | 45,250,961 | 45,263,301 | $3.09\times{10}^{-17}$ | $1.89\times{10}^{-14}$ |
| *BCAM* | 19 | 45,312,337 | 45,324,677 | $3.35\times{10}^{-13}$ | $1.47\times{10}^{-10}$ |
| *PVRL2* | 19 | 45,349,392 | 45,392,485 | $7.07\times{10}^{-23}$ | $5.85\times{10}^{-20}$ |
| *TOMM40 ^a,b^* | 19 | 45,394,476 | 45,406,935 | $1.52\times{10}^{-69}$ | $7.13\times{10}^{-66}$ |
| *APOE* | 19 | 45,408,955 | 45,412,650 | $2.59\times{10}^{-13}$ | $1.22\times{10}^{-10}$ |
| *APOC1* | 19 | 45,417,920 | 45,422,606 | $7.83\times{10}^{-12}$ | $3.16\times{10}^{-9}$ |
| *CLPTM1 ^a,b^* | 19 | 45,457,847 | 45,496,598 | $1.48\times{10}^{-30}$ | $1.60\times{10}^{-27}$ |
| *RELB ^a^* | 19 | 45,504,694 | 45,541,452 | $7.48\times{10}^{-41}$ | $1.50\times{10}^{-37}$ |
| *CLASRP ^a,b^* | 19 | 45,542,297 | 45,574,214 | $1.91\times{10}^{-90}$ | $2.69\times{10}^{-86}$ |
| *ZNF296 ^b^* | 19 | 45,574,758 | 45,579,845 | $4.05\times{10}^{-16}$ | $2.28\times{10}^{-13}$ |
| *GEMIN7* | 19 | 45,582,529 | 45,594,782 | $1.56\times{10}^{-16}$ | $9.17\times{10}^{-14}$ |
| *PPP1R37* | 19 | 45,595,049 | 45,651,335 | $1.02\times{10}^{-17}$ | $6.49\times{10}^{-15}$ |
| *NKPD1* | 19 | 45,653,007 | 45,663,408 | $2.61\times{10}^{-19}$ | $1.84\times{10}^{-15}$ |
| *TRAPPC6A ^a,b^* | 19 | 45,666,186 | 45,681,485 | $5.33\times{10}^{-51}$ | $1.50\times{10}^{-47}$ |
| *BLOC1S3* | 19 | 45,682,002 | 45,685,057 | $6.28\times{10}^{-5}$ | $1.67\times{10}^{-2}$ |
| *MARK4 ^a,b^* | 19 | 45,754,549 | 45,808,541 | $3.16\times{10}^{-71}$ | $2.22\times{10}^{-67}$ |
| *ERCC2* | 19 | 45,854,245 | 45,873,876 | $4.76\times{10}^{-5}$ | $1.31\times{10}^{-2}$ |
| *PPP1R13L ^a^* | 19 | 45,882,891 | 45,909,607 | $1.25\times{10}^{-37}$ | $1.95\times{10}^{-34}$ |
| *CD3EAP ^b^* | 19 | 45,909,466 | 45,914,024 | $1.15\times{10}^{-7}$ | $4.15\times{10}^{-5}$ |
| *ERCC1* | 19 | 45,910,590 | 45,982,086 | $6.89\times{10}^{-14}$ | $3.34\times{10}^{-11}$ |
| *FOSB* | 19 | 45,971,252 | 45,978,414 | $4.06\times{10}^{-21}$ | $3.17\times{10}^{-18}$ |
| *RTN2 ^b^* | 19 | 45,988,549 | 46,000,313 | $1.89\times{10}^{-26}$ | $1.78\times{10}^{-23}$ |
| *PPM1N ^b^* | 19 | 45,992,034 | 46,005,768 | $1.30\times{10}^{-15}$ | $7.04\times{10}^{-13}$ |
| *VASP* | 19 | 46,010,687 | 46,030,236 | $3.11\times{10}^{-41}$ | $7.28\times{10}^{-38}$ |
| *GPR4* | 19 | 46,093,024 | 46,105,466 | $8.88\times{10}^{-6}$ | $2.84\times{10}^{-3}$ |
| *EML2 ^a,b^* | 19 | 46,112,659 | 46,148,726 | $1.83\times{10}^{-34}$ | $2.35\times{10}^{-31}$ |
| *GIPR ^a,b^* | 19 | 46,171,501 | 46,185,704 | $4.21\times{10}^{-25}$ | $3.70\times{10}^{-22}$ |
| *SNRPD2 ^b^* | 19 | 46,190,712 | 46,195,443 | $9.97\times{10}^{-15}$ | $5.19\times{10}^{-12}$ |
| *QPCTL* | 19 | 46,195,740 | 46,207,240 | $7.87\times{10}^{-12}$ | $3.16\times{10}^{-9}$ |
| *FBXO46 ^a,b^* | 19 | 46,213,886 | 46,234,151 | $1.74\times{10}^{-36}$ | $2.45\times{10}^{-33}$ |
| *DMPK* | 19 | 46,272,977 | 46,285,815 | $6.79\times{10}^{-32}$ | $7.95\times{10}^{-29}$ |
| *DMWD* | 19 | 46,286,204 | 46,296,060 | $1.40\times{10}^{-4}$ | $3.57\times{10}^{-2}$ |
| *SYMPK* | 19 | 46,318,692 | 46,366,548 | $1.54\times{10}^{-4}$ | $3.87\times{10}^{-2}$ |
| *IRF2BP1 ^b^* | 19 | 46,386,865 | 46,389,376 | $5.02\times{10}^{-39}$ | $8.83\times{10}^{-36}$ |
| *MYPOP ^b^* | 19 | 46,393,284 | 46,405,862 | $2.80\times{10}^{-13}$ | $1.27\times{10}^{-10}$ |
| *NLRP2* | 19 | 55,476,437 | 55,512,510 | $2.82\times{10}^{-5}$ | $8.09\times{10}^{-3}$ |
| *HAR1A* | 20 | 61,733,556 | 61,735,738 | $1.17\times{10}^{-4}$ | $3.05\times{10}^{-2}$ |

1. Genes also identified as significant by VC-TWAS with individual-level GWAS data of ROS/MAP and Mayo Clinic cohorts.
2. Genes identified as significant by both VC-TWAS and Burden-TWAS using IGAP summary statistics with filtered cis-eQTL DPR weights.
