# Supplementary material for: Novel Variance-Component TWAS method for studying complex human diseases with applications to Alzheimer’s dementia
Source: PLoS Genet. 2021 Apr 2;17(4):e1009482. doi: 10.1371/journal.pgen.1009482 (PMC8046351; doi:10.1371/journal.pgen.1009482)
Supplement: S4 Table — (DOCX) [file pgen.1009482.s016.docx]

**S4 Table:** Novel significant genes identified by VC-TWAS using summary statistics with BGW weights on IGAP summary statistics.

| **Gene Name** | **CHROM** | **Start** | **End** | **P-value** | **FDR** |
| --- | --- | --- | --- | --- | --- |
| PPIEL | 1 | 39,997,509 | 40,024,379 | $1.41\times{10}^{-4}$ | $3.01\times{10}^{-2}$ |
| ARHGAP29 | 1 | 94,614,543 | 94,740,624 | $1.14\times{10}^{-7}$ | $6.11\times{10}^{-5}$ |
| RWDD3 | 1 | 95,699,710 | 95,712,781 | $4.17\times{10}^{-53}$ | $2.94\times{10}^{-49}$ |
| GPATCH2 | 1 | 217,600,333 | 217,804,424 | $2.41\times{10}^{-7}$ | $1.17\times{10}^{-4}$ |
| RP11-211A18.2 | 1 | 227,421,969 | 227,423,056 | $4.84\times{10}^{-6}$ | $1.59\times{10}^{-3}$ |
| C2orf50 | 2 | 11,273,178 | 11,286,916 | $4.92\times{10}^{-5}$ | $1.20\times{10}^{-2}$ |
| ANKRD30BL | 2 | 132,905,163 | 133,015,542 | $1.43\times{10}^{-8}$ | $1.06\times{10}^{-5}$ |
| TTC21B | 2 | 166,713,984 | 166,810,353 | $4.86\times{10}^{-7}$ | $2.14\times{10}^{-4}$ |
| SPEG | 2 | 220,299,567 | 220,363,009 | $1.11\times{10}^{-10}$ | $1.12\times{10}^{-7}$ |
| FBXO36 | 2 | 230,787,017 | 230,877,825 | $3.14\times{10}^{-9}$ | $2.60\times{10}^{-6}$ |
| PTH1R | 3 | 46,919,235 | 46,945,287 | $1.52\times{10}^{-6}$ | $5.50\times{10}^{-4}$ |
| ZXDC | 3 | 126,156,443 | 126,194,762 | $7.11\times{10}^{-6}$ | $2.13\times{10}^{-3}$ |
| XPO5 | 6 | 43,490,071 | 43,543,812 | $3.76\times{10}^{-8}$ | $2.40\times{10}^{-5}$ |
| RP1-180E22.3 | 6 | 52,442,282 | 52,444,325 | $2.14\times{10}^{-4}$ | $4.30\times{10}^{-2}$ |
| BCLAF1 | 6 | 136,578,000 | 136,610,989 | $6.31\times{10}^{-7}$ | $2.69\times{10}^{-4}$ |
| AC017116.8 | 7 | 44,078,769 | 44,081,905 | $6.18\times{10}^{-8}$ | $3.70\times{10}^{-5}$ |
| MRPL15 | 8 | 55,047,769 | 55,060,461 | $6.21\times{10}^{-5}$ | $1.43\times{10}^{-2}$ |
| VCPIP1 | 8 | 67,540,721 | 67,579,452 | $3.52\times{10}^{-9}$ | $2.75\times{10}^{-6}$ |
| GEM | 8 | 95,261,480 | 95,274,578 | $5.51\times{10}^{-5}$ | $1.31\times{10}^{-2}$ |
| *TRAF1* | 9 | 123,664,670 | 123,691,451 | $5.69\times{10}^{-21}$ | $1.00\times{10}^{-17}$ |
| PBX3 | 9 | 128,509,623 | 128,729,656 | $1.23\times{10}^{-6}$ | $4.56\times{10}^{-4}$ |
| C9orf167 | 9 | 140,172,200 | 140,177,093 | $5.66\times{10}^{-6}$ | $1.81\times{10}^{-3}$ |
| AGAP10 | 10 | 47,191,843 | 47,239,738 | $1.29\times{10}^{-5}$ | $3.64\times{10}^{-3}$ |
| HELLS | 10 | 96,305,546 | 96,373,662 | $3.51\times{10}^{-21}$ | $7.05\times{10}^{-18}$ |
| PKD2L1 | 10 | 102,047,902 | 102,090,243 | $1.03\times{10}^{-60}$ | $1.45\times{10}^{-56}$ |
| C10orf137 | 10 | 127,408,083 | 127,452,712 | $3.26\times{10}^{-6}$ | $1.09\times{10}^{-3}$ |
| SLC39A13 | 11 | 47,428,682 | 47,438,047 | $1.81\times{10}^{-4}$ | $3.76\times{10}^{-2}$ |
| LETMD1 | 12 | 51,441,744 | 51,454,207 | $6.19\times{10}^{-6}$ | $1.94\times{10}^{-3}$ |
| ZBTB39 | 12 | 57,392,617 | 57,400,230 | $8.73\times{10}^{-5}$ | $1.95\times{10}^{-2}$ |
| KITLG | 12 | 88,885,884 | 88,974,628 | $1.74\times{10}^{-4}$ | $3.66\times{10}^{-2}$ |
| NT5DC3 | 12 | 104,164,230 | 104,234,975 | $3.95\times{10}^{-12}$ | $4.28\times{10}^{-9}$ |
| SERTM1 | 13 | 37,248,048 | 37,271,976 | $1.97\times{10}^{-4}$ | $4.03\times{10}^{-2}$ |
| SEC23A | 14 | 39,501,122 | 39,578,850 | $8.85\times{10}^{-5}$ | $1.95\times{10}^{-2}$ |
| KTN1-AS1 | 14 | 55,965,995 | 56,046,828 | $1.09\times{10}^{-6}$ | $4.25\times{10}^{-4}$ |
| ACOT4 | 14 | 74,058,409 | 74,063,200 | $1.30\times{10}^{-34}$ | $6.10\times{10}^{-31}$ |
| RP13-487P22.1 | 15 | 25,590,779 | 25,592,382 | $3.82\times{10}^{-5}$ | $9.60\times{10}^{-3}$ |
| C15orf58 | 15 | 90,777,039 | 90,785,315 | $1.17\times{10}^{-7}$ | $6.11\times{10}^{-5}$ |
| IGSF6 | 16 | 21,652,608 | 21,663,981 | $1.37\times{10}^{-13}$ | $1.75\times{10}^{-10}$ |
| AC012146.7 | 17 | 5,014,762 | 5,018,299 | $5.58\times{10}^{-20}$ | $8.74\times{10}^{-17}$ |
| AC092296.1 | 19 | 36,804,643 | 36,822,602 | $2.24\times{10}^{-13}$ | $2.63\times{10}^{-10}$ |
| GNAS | 20 | 57,414,772 | 57,486,247 | $8.07\times{10}^{-25}$ | $2.27\times{10}^{-21}$ |
| FOXRED2 | 22 | 36,883,236 | 36,903,148 | $1.05\times{10}^{-6}$ | $4.24\times{10}^{-4}$ |
| H1F0 | 22 | 38,201,113 | 38,203,442 | $8.26\times{10}^{-5}$ | $1.88\times{10}^{-2}$ |
| PPPDE2 | 22 | 41,994,031 | 42,017,100 | $4.15\times{10}^{-10}$ | $3.65\times{10}^{-7}$ |
| PPP6R2 | 22 | 50,781,732 | 50,883,514 | $6.97\times{10}^{-21}$ | $4.77\times{10}^{-2}$ |
