# Supplementary material for: Novel Variance-Component TWAS method for studying complex human diseases with applications to Alzheimer’s dementia
Source: PLoS Genet. 2021 Apr 2;17(4):e1009482. doi: 10.1371/journal.pgen.1009482 (PMC8046351; doi:10.1371/journal.pgen.1009482)
Supplement: S5 Table — (DOCX) [file pgen.1009482.s017.docx]

**S5 Table:** Pros and cons of existing popular TWAS methods.

| **Methods** | **GReX Model** | **Association Test** | **Pros** | | **Cons** | |
| --- | --- | --- | --- | --- | --- | --- |
| PrediXcan | Two-Stage Elastic-Net Regression | Burden Test (Burden-TWAS) | Computationally Efficient | | Fail to Account for eQTL Effect Size Estimation Uncertainty  Linear Assumption between SNP Effects on Transcriptome and Phenotype | Obtained Fewer Genes with Significant GReX Prediction Models |
| TIGAR | Two-Stage Bayesian DPR Regression |  | Model GReX by a Nonparametric Bayesian DPR Model | |  | Reduced Power for Testing Sparse Causal SNPs |
| FUSION | Two-Stage BSLMM Regression |  | Model GReX by a Bayesian Sparse Linear Mixed Model | |  | Computationally Expensive for Training GReX Prediction Model; |
| BGW-TWAS | Two-Stage BVSR Considering both Cis- and Trans- eQTL |  | Modeling Genome-wide Cis- and Trans- eQTL Data by BVSR | |  |  |
| CoMM /  CoMM-SS | Jointly Model Reference and Test Cohorts by Linear Mixed Regression | Likelihood Ratio Test | Account for eQTL Effect Size Estimation Uncertainty | Test for Random Effect | Computationally Expensive with Thousands of Test SNPs | Derived Only for Testing Continuous Phenotype |
| PMR-Egger | Jointly Model Reference and Test Cohorts by Linear Regression |  |  | Equivalent to Mendelian Randomization |  |  |
| moPMR-Egger | Jointly Model Reference and Test Cohorts by Linear Regression with Multiple Phenotypes |  |  | Test Multiple Phenotypes |  |  |
| VC-TWAS | Two-Stage Bayesian DPR Regression | Variance Component Test | Relax Linear Relationship Assumption between SNP Effects on Transcriptome and Phenotype | | Computationally More Expensive than Burden Test | Reduced Power Compared to Jointly Modeling Reference and Test Cohorts |
