# Supplementary material for: Novel Variance-Component TWAS method for studying complex human diseases with applications to Alzheimer’s dementia
Source: PLoS Genet. 2021 Apr 2;17(4):e1009482. doi: 10.1371/journal.pgen.1009482 (PMC8046351; doi:10.1371/journal.pgen.1009482)
Supplement: Attachment — Submitted filename: Review.pdf [file pgen.1009482.s018.pdf]

This paper proposes to apply the SKAT model to infer the association between cis-eQTLs and phenotype, with variances of SNP effects being linear in the eQTL effect sizes square. I think it would be helpful if the motivation to use the SKAT model can be explained in more details. Since testing $\tau = 0$ is equivalent to testing $\boldsymbol{\beta} = \mathbf{0}$, a natural questions to ask is why not testing $\boldsymbol{\beta} = \mathbf{0}$ directly using F-test for example, given that F-test is computationally simple and fast? SKAT tries to deal with rare variant, what about VC-TWAS? How much gain in power does VC-TWAS enjoy if not in the rare variant case?

It is mentioned that "our novel VC-TWAS provides an important analytic tool for identifying risk genes whose effects on phenotypes are mediated through transcriptomes." The existence of cis-association and association between the cis-eQTLs and phenotype does not guarantee that. The relationship between the gene expression level and the phenotype can be simply a result of shared variants. Under what assumptions can VC-TWAS identify genes affecting phenotype?

A minor point, is it an incomplete sentence? Lines 434-435: VC-TWAS with PrediXcan weights, no significant genes were identified with FDR <0.05 (S5-7 Figs), neither the genes with smallest p-values were proximal to APOE.
