# Supplementary material for: Novel Variance-Component TWAS method for studying complex human diseases with applications to Alzheimer’s dementia
Source: PLoS Genet. 2021 Apr 2;17(4):e1009482. doi: 10.1371/journal.pgen.1009482 (PMC8046351; doi:10.1371/journal.pgen.1009482)
Supplement: Attachment — Submitted filename: review.docx [file pgen.1009482.s019.docx]

This paper proposes a novel Variance-Component TWAS procedure (VC-TWAS) that assumes the effects of cis-eQTL SNPs on phenotype are random (with variance proportional to corresponding cis-eQTL effects in reference dataset) rather than fixed. They further applied VC-TWAS using cis-eQTL effect sizes estimated by a nonparametric Bayesian method to study Alzheimer’s dementia (AD) related phenotypes and detected 13 genes significantly associated with AD, including 6 known GWAS risk loci such as TOMM40. It is an interesting problem that authors propose variance component tests to perform TWAS. Here are my major comments.

1. First, I think authors should compare other alternative methods in TWAS as there are several methods that may outperform standard TWAS, e.g., [1] and [2]. Authors need to numerically compare VC-TWAS these methods as well. Particularly, [1] connects CoMM with other SKAT and PrediXcan, showing how the improvement comes from (Section 2.4 in [1]). Since the test for equation (8) is closely related to equation (14) in [1], authors should cite the relevant reference for their works.
2. In the simulation studies, what are the sample sizes for equation (9)? As sample sizes for eqtl studies increase, the difference between PrediXcan and other refined ones becomes similar. In addition, Table 1 that summarizes the type I error is not enough especially for genome-wide scan. qq-plots for null simulations are more appropriate for type I error control.
3. In abstract, it is too strong to state that VC-TWAS gain > 2X power. This can be only done by simulation studies with some favorable settings. I am wondering why authors did not apply alternative methods to the real data analysis. The comparison in the real data analysis will be more interesting.

[1] Yang C, Wan X, Lin X, et al. CoMM: a collaborative mixed model to dissecting genetic contributions to complex traits by leveraging regulatory information[J]. Bioinformatics, 2019, 35(10): 1644-1652.

[2] Nagpal S, Meng X, Epstein M P, et al. TIGAR: an improved Bayesian tool for transcriptomic data imputation enhances gene mapping of complex traits[J]. The American Journal of Human Genetics, 2019, 105(2): 258-266.
