# Supplementary material for: Novel Variance-Component TWAS method for studying complex human diseases with applications to Alzheimer’s dementia
Source: PLoS Genet. 2021 Apr 2;17(4):e1009482. doi: 10.1371/journal.pgen.1009482 (PMC8046351; doi:10.1371/journal.pgen.1009482)
Supplement: Attachment — Submitted filename: ResponseToReviewers.docx [file pgen.1009482.s020.docx]

**Response to Reviewers**

We are grateful for the reviewers’ helpful comments, which has enhanced the quality of our manuscript. In addition to addressing all reviewers’ comments, we enhanced the novelty of our VC-TWAS approach by modifying the framework to enable analyses based only on GWAS summary statistics if individual-level data are not available. We applied our VC-TWAS method to the public IGAP summary statistics of the GWAS of Alzheimer’s disease (AD) with sample size ~54K. The IGAP results not only validated our findings with the individual-level GWAS data of ROS/MAP/Mayo samples, but also identified additional potential risk genes for AD.

**Reviewer #1**

This paper proposes to apply the SKAT model to infer the association between cis-eQTLs and phenotype, with variances of SNP effects being linear in the eQTL effect sizes square.

1. I think it would be helpful if the motivation to use the SKAT model can be explained in more details. Since testing τ=0 is equivalent to testing 𝛃=𝟎, a natural question to ask is why not testing 𝛃=𝟎 directly using F-test for example, given that F-test is computationally simple and fast?

We thank the reviewer for this comment. The distribution of the traditional F-test assumes degrees of freedom that are equal to the number of SNPs that are jointly modeled within the gene. Thus, the F-test assumes that all the modeled SNPs within the gene are independent of one another. This assumption is doubtful to hold in practice because of likely correlation among variants due to widespread linkage disequilibrium (LD) within the gene. This LD will result in the ‘effective’ number of degrees of freedom being smaller than the number assumed by the F-test, which will lead to a less powerful test as a result (Kwee et al. 2008; Tzeng and Zhang 2007). Our VC-TWAS, on the other hand, empirically accounts for the LD among variants in the gene when deriving the distribution of the test under the null and thus remains powerful in this situation.

We note that there is a variation of a F-test for multi-SNP association analysis called genomic distance-based regression (GDBR) developed by Wessel and Schork (2006) that accounts for LD among variants. Pan (2011) showed GDBR has a similar form to the kernel framework we implement in VC-TWAS. However, while our VC-TWAS framework can derive p-values rapidly based on asymptotic theory, GDBR requires permutations to establish significance and thus is impractical to scale for genome-wide TWAS.

Kwee LC, Liu D, Lin X, Ghosh D, Epstein MP. A powerful and flexible multi-locus association test for quantitative traits. Am. J. Hum. Genet. 2008;82:386–397

Pan W. Relationship between genomic distance-based regression and kernel machine regression for multi-marker association testing. Genetic Epidemiology 2011; 35: 2011-2016

Tzeng J-Y, Zhang D. Haplotype-based association analysis via variance-components score test. Am J Hum Genet. 2007;81:927–938

Wessel J, Schork NJ. Generalized genomic distance-based regression methodology for multilocus association analysis. Am J Hum Genet. 2006;79:792–806

2. SKAT tries to deal with rare variant, what about VC-TWAS? How much gain in power does VC-TWAS enjoy if not in the rare variant case?

We thank the reviewer for this comment. We note that both SKAT and VC-TWAS are actually derived from the same analytic framework of least-square kernel-machine regression (Liu et al. 2017). The original SKAT framework was actually developed to test for multiple common SNPs in a gene (Kwee et al. 2008; Wu et al. 2010) with SNPs weighted by minor-allele frequency (MAF); the popular rare-variant version (Wu et al. 2011) was not developed until a couple years later. Our VC-TWAS method performs TWAS by taking the original version of SKAT for common SNPs and changing the SNP weights such that, instead of using weights based on MAF, we instead use weights based on squared cis-eQTL effect sizes from a reference transcriptomic data set.

To illustrate the power gain of VC-TWAS over traditional SKAT method, we applied the SKAT method for common SNPS to one of our simulation scenarios and compared the power between the two approaches. As shown in the following Figure 1, we can see that under Model 1 (which met the assumptions of Burden-TWAS methods), both VC-TWAS methods (as well as Burden-TWAS methods) had higher power than SKAT. Under Model 2 (where the assumptions by Burden-TWAS was violated), we note that VC-TWAS nearly universally outperformed SKAT. Interestingly, we do notice that SKAT does outperform traditional Burden-TWAS under Model II, highlighting again the power loss of using Burden-TWAS when the method’s modeling assumptions do not hold.

Figure 1. Compare TWAS methods with SKAT using default SNP weights based on minor allele frequency in the simulation study with $p_{causal}=0.2$ (the same scenario as shown in Figure 1 in the manuscript).

Kwee LC, Liu D, Lin X, Ghosh D, Epstein MP. A powerful and flexible multilocus association test for quantitative traits. Am. J. Hum. Genet. 2008;82:386–397

Liu D, Lin X, Ghosh D. Semiparametric regression of multidimensional genetic pathway data: Least squares kernel machines and linear mixed models. Biometrics 2007; 63:1079-1088

Wu MC, Kraft P, Epstein MP, Taylor DM, Chanock SJ, Hunter DJ, Lin X. Powerful SNP-Set Analysis for Case-Control Genome-wide Association Studies. Am J Hum Genet. 2010;86:929–942

Wu MC, Lee S, Cai T, Li Y, Boehnke M, Lin X. Rare-variant association testing for sequencing data with the sequence kernel association test. Am J Hum Genet. 2011; 89: 82-93

3. It is mentioned that “our novel VC-TWAS provides an important analytic tool for identifying risk genes whose effects on phenotypes are mediated through transcriptomes.” The existence of cis-association and association between the cis-eQTLs and phenotype does not guarantee that. The relationship between the gene expression level and the phenotype can be simply a result of shared variants. Under what assumptions can VC-TWAS identify genes affecting phenotype?

We appreciate reviewer’s comment about this. We agree that this statement did not accurately describe all possibilities. We have modified this in the manuscript.

4. A minor point, is it an incomplete sentence? Lines 434-435: VC-TWAS with PrediXcan weights, no significant genes were identified with FDR <0.05 (S5-7 Figs), neither the genes with smallest p-values were proximal to APOE.

Thanks a lot for pointing this out. We have proof read the main text and corrected this sentence.

**Reviewer #2**:

This paper proposes a novel Variance-Component TWAS procedure (VC-TWAS) that assumes the effects of cis-eQTL SNPs on phenotype are random (with variance proportional to corresponding cis-eQTL effects in reference dataset) rather than fixed. They further applied VC-TWAS using cis-eQTL effect sizes estimated by a nonparametric Bayesian method to study Alzheimer’s dementia (AD) related phenotypes and detected 13 genes significantly associated with AD, including 6 known GWAS risk loci such as TOMM40. It is an interesting problem that authors propose variance component tests to perform TWAS. Here are my major comments.

1. First, I think authors should compare other alternative methods in TWAS as there are several methods that may outperform standard TWAS, e.g., [1] and [2]. Authors need to numerically compare VC-TWAS these methods as well. Particularly, [1] connects CoMM with other SKAT and PrediXcan, showing how the improvement comes from (Section 2.4 in [1]). Since the test for equation (8) is closely related to equation (14) in [1], authors should cite the relevant reference for their works.

We want to thank the Reviewer 2 for this suggestion. We did compare VC-TWAS with TIGAR TWAS methods (reference [2] mentioned by the Reviewer) in our initial submission. We had denoted this TIGAR TWAS method as Burden-TWAS using DPR weights in our manuscript. We also compared VC-TWAS with PrediXcan, which we denote as Burden-TWAS using PrediXcan weights in our manuscript.

In this revision, we have cited the CoMM method papers in our Introduction section, and compared with CoMM (reference [1] mentioned by the Reviewer) and the recent version of CoMM_S2 using GWAS summary statistics (recently published in *Bioinformatics (*https://pubmed.ncbi.nlm.nih.gov/31755899/*)*) in one of our simulation scenarios. These results are included in our Simulation Results section (Page 15, lines 358-365 in the tracked version) and Fig S2. We show that the CoMM method indeed is very powerful for all simulation models. However, VC-TWAS has two considerable advantages over CoMM. First, by design, CoMM is only applicable to TWAS of continuous traits. VC-TWAS, on the other hand, is applicable to both continuous traits and binary traits. Second, CoMM is computationally demanding to run as it required ~30 minutes per test in our simulation studies. In comparing CoMM in one of our simulation scenarios, we observed the CoMM cost 400 CPU hours to run. Compared to CoMM, VC-TWAS is much more efficient to run. When considering our simulated datasets, our method was ~262 to ~600 times faster (depending on gene size) than CoMM when using individual-level data and ~14 to ~265 times faster (depending on gene size) when using summary-level data (see Table S1).

2. In the simulation studies, what are the sample sizes for equation (9)? As sample sizes for eqtl studies increase, the difference between PrediXcan and other refined ones becomes similar.

In our simulation studies, the training sample size used to estimate the cis-eQTL weights was 499, while the test sample size was 1232. We based these numbers off those observed in the ROS/MAP study. In this revision, we performed power analysis with a few different test sample sizes (400, 800, 1232), which showed that all TWAS methods had increased power as the test sample size increased.

3. In addition, Table 1 that summarizes the type I error is not enough especially for genome-wide scan. qq-plots for null simulations are more appropriate for type I error control.

The number of total null simulation replicates for evaluating type I error is subject to the total computation time. We think our Table 1 shows that all methods controlled Type I error well with respect to the genome-wide significance threshold of gene-based tests. In this revision, we provide the qq-plot of all null simulations in Fig S3, which shows that our null p-values are well calibrated.

4. In abstract, it is too strong to state that VC-TWAS gain > 2X power. This can be only done by simulation studies with some favorable settings. I am wondering why authors did not apply alternative methods to the real data analysis. The comparison in the real data analysis will be more interesting.

We want to thank the Reviewer 2 for pointing this out. We have clarified our statement in the abstract. We have applied both PrediXan and TIGAR methods to the same ROS/MAP real data in our previously published TIGAR paper (Nagpal S. et. al. AJHG, 2019; reference [2] provided by Reviewer 2). We observed that VC-TWAS still identified more number of significant genes nearby the major know AD risk gene *APOE*.

[1] Yang C, Wan X, Lin X, et al. CoMM: a collaborative mixed model to dissecting genetic contributions to complex traits by leveraging regulatory information[J]. Bioinformatics, 2019, 35(10): 1644-1652.

[2] Nagpal S, Meng X, Epstein M P, et al. TIGAR: an improved Bayesian tool for transcriptomic data imputation enhances gene mapping of complex traits[J]. The American Journal of Human Genetics, 2019, 105(2): 258-266.

**Reviewer #3**:

Tang et al. proposed a TWAS method following the idea of the gene-based variance component test SKAT with cis-variant weights trained with Dirichlet process regression (DPR). The idea of combining TWAS and SKAT is novel, and the applications on Alzheimer’s disease data are interesting. The manuscript is also clearly written. Below I list some comments that may further improve the paper.

Major comments:
“As DPR method produces non-zero cis-eQTL effect size estimates for almost all SNPs within a test gene region (with most cis-eQTL effect size estimates being close to zero [8]), we explored an alternate VC-TWAS that considered a reduced set of SNPs by filtering out those with cis-eQTL effect size estimates smaller than the median cis-eQTL effect size estimate.”
1) Is the median cis-eQTL effect size estimate for each gene or all genes?

For the reason of using the same threshold to filter eQTL effect sizes for all genes, we chose to use 1e-4 which kept approximately half of the SNPs for VC-TWAS test per gene. As shown in the Fig S4, we show the box plot of the SNP number per gene for all genome-wide genes, when using the cis-eQTL DPR weights trained with the real ROS/MAP data. In our simulation studies, we used the median of the median cis-eQTL effect size per simulation from a total of 1000 simulation replicates per scenario.

2) Other than using this arbitrary threshold for filtering, a method BGW-TWAS developed earlier by the authors’ group adopts a spike-and-slab prior that is commonly used in Bayesian variable selection. I am wondering what the performance of DPR as compared to the spike-and-slab prior. Additionally, BGW-TWAS considers both cis and trans- eQTLs and has trained the same datasets as this manuscript. It would be interesting to use the BGW-TWAS trained cis- and trans- eQTL weights in this VC-TWAS approach. This will help compare: i) DPR vs. the spike-and-slab prior, and ii) cis- only vs. both cis- and trans- eQTLs.

We want to thank the Reviewer 3 for this helpful suggestion. In this revision, we applied VC-TWAS with the cis- and trans- eQTL weights obtained by the BGW-TWAS method that used the spike-and-slab prior. Comparing to the results by VC-TWAS using cis-eQTL DPR weights (Fig 3), we found that VC-TWAS using BGW weights that considered trans-eQTL effects identified the same risk genes on chromosome 19 identified previously using just cis-eQTL weights but also identified an additional group of potential risk genes (see Fig 4). We list these additional genes in Table S4.

The results by Burden-TWAS using BGW weights (cis-only, cis- and trans-) with the same application data set were provided in our previously published BGW-TWAS paper (Lumingham J., et. al. AJHG 2020). We observed that besides the significant locus on chromosome 19 near APOE that were identified by all TWAS approaches, the VC-TWAS using both cis- and trans- BGW weights identified more number of novel risk genes of AD than the existing Burden-TWAS approach.

As written in the Results section, “We detected total 71 significant genes with FDR < 0.05 (Fig 3B; Table S4; Fig S11B), among which 6 genes were identified by GWAS and 22 genes were shown to be related with AD or other neurological diseases by previous studies and (Table 3).”

4) Given that the main contribution of this manuscript is to use different SNP weights, I am curious about the real data results using the original SKAT to see the gain by using this new weighting via TWAS.

Please see our response to the second comment by Reviewer 1 for the rational of using the SKAT framework and power improvement over the original SKAT.

“Mayo Clinic LOAD GWAS data contain samples from two clinical AD Case-Control series:
Mayo Clinic Jacksonville (MCJ: 353 AD cases and 331 Controls), Mayo Clinic Rochester (MCR: 291 AD cases and 787 Controls) and a neuropathological series of autopsy-confirmed subjects from the Mayo Clinic Brain Bank (MCBB: 298 AD cases and 223 non-AD Controls) [24, 25]. In total, we have 844 cases with LOAD and 1,255 controls without a dementia diagnosis.”
1) This part was from the data description on the Synapse website, but the sum of the sample size of the three cohorts does not equal to the total number listed above. Specifically, the sample size of MCR and AD cases of MCBB does not match with those reported in Allen et al. Scientific Data 2016.

We want to thank the Reviewer 3 for catching this. The data description of sample sizes was wrong on the Synapse website where we downloaded the data. The total number of cases and controls stated in our manuscript is correct with our downloaded data. We obtained the correct sample size for each sub-study from the original Mayo Clinic GWAS paper. These numbers are now corrected in this revision (page 10 lines 235-242 in the tracked version).

“Mayo Clinic LOAD GWAS data contain samples from two clinical AD Case-Control series: Mayo Clinic Jacksonville (MCJ: 353 AD cases and 331 Controls), Mayo Clinic Rochester (MCR: 245 AD cases and 701 Controls) and a neuropathological series of autopsy-confirmed subjects from the Mayo Clinic Brain Bank (MCBB: 246 AD cases and 223 non-AD Controls)”

2) In the real data analysis of Mayo Clinic data, the authors probably need to adjust for the cohort variable. MCJ and MCR samples are antemortem, but MCBB samples are postmortem.

We agree with the Reviewer’s comment. Unfortunately, Synapse did not provide the study indexes in the Mayo Clinic data that we downloaded, such that we can’t adjust for the cohort variable. Instead, we provide the qq-plot of all VC-TWAS results with the real data in Fig S6, which shows VC-TWAS analyses of the Mayo Clinic data are well calibrated.

It is not clear if the outcome/AD definition is the same across ROSMAP data and the three cohorts of Mayo Clinic data. Below is an excerpt on the AD definition of Mayo data copied from the Synapse website.
“All subjects from the clinical series (MCJ and MCR) were diagnosed by a Mayo Clinic neurologist; all control subjects had a Clinical Dementia Rating score of zero at the most recent time of testing; all LOAD patients had a diagnosis of probable or possible AD according to the NINCDS-ADRDA criteria. Subjects selected from the Mayo Clinic Brain Bank underwent neuropathologic evaluation by Dr. Dennis Dickson. All ADs had definite diagnosis according to the NINCDS-ADRDA criteria and had Braak scores of ≥4.0. All non–AD Controls had Braak scores of ≤2.5; many had brain pathology unrelated to AD and, in particular, many of the non-AD samples used in the brain expression data were selected based on the presence of primary tauopathy (progressive supranuclear palsy).”

1) Were all AD patients in both ROSMAP and Mayo LOAD GWAS data diagnosed by a neurologist with the same criterion?

For the ROSMAP cohort, a clinical diagnosis of cognitive status is rendered at every assessment based on a three-stage process including computer scoring of cognitive tests, clinical judgment by a neuropsychologist, and diagnostic classification by a clinician. All participants undergo a uniform, structured, clinical evaluation including a battery of 19 cognitive tests. These tests were scored by computer using a decision tree designed to mimic clinical judgment and a rating of severity of impairment was given for 5 cognitive domains. A neuropsychologist, blinded to participant demographics, reviews the impairment ratings and other clinical information and renders a clinical judgment regarding the presence of impairment and dementia. A clinician (neurologist, geriatrician, or geriatric nurse practitioner) then reviews all available data and examines the participant and renders a final diagnostic classification.

Clinical diagnosis of dementia and clinical Alzheimer’s dementia are based on criteria of the joint working group of the National Institute of Neurological and Communicative Disorders and Stroke and the Alzheimer’s Disease and Related Disorders Association (NINCDS/ADRDA). The diagnosis of Alzheimer’s dementia requires evidence of a meaningful decline in cognitive function relative to a previous level of performance with impairment in memory and at least one other area of cognition.

Even though the same NINCDS-ADRDA criteria was used to make clinical AD diagnosis for all cohorts, different additional criteria were used for MCJ/MCR, MCBB, and ROS/MAP. We think this should not affect the main meta VC-TWAS results, which were further replicated in our studies using the IGAP GWAS summary data of AD.

2) Even if 1) is true, the diagnosis of subjects in Mayo data has been assisted with NINCDS-ADRDA criteria and Braak score. For instance, “all LOAD patients had a diagnosis of probable or possible AD according to the NINCDS-ADRDA criteria.” Is this true for ROSMAP data?

Yes. The same NINCDS-ADRDA criterion was also used in ROSMAP data.

“All MCBB ADs had definite diagnosis according to the NINCDS-ADRDA criteria and had Braak scores of ≥4.0, all non–AD Controls had Braak scores of ≤2.5” is this true for all other cohorts?

I know it may be hard to check this, but making sure each dataset has the same AD definition and matched other clinical variables would facilitate the interpretation of the meta-analysis.

The Braak score might only be used for the MCBB samples. As we explained in the response to the first comment by Reviewer 3, our meta VC-TWAS results using individual-level GWAS data of ROS/MAP and Mayo Clinic cohorts were all replicated with the IGAP that have a much larger sample size.

3) The non-AD controls in MCBB have other pathologies. Is this true for other cohorts? Would this cofound the comparison as well?

Before meta-analysis is done, are there any overlapping signals between the two data cohorts: ROSMAP and Mayo LOAD GWAS? This would help check the reproducibility of results.

We want to thank the reviewer for this suggestion. ROS/MAP cohort profiled other types of brain pathologies. We think it is less likely to be confounded by other types of pathologies as beta-amyloid and tangles are two main pathology for AD. Unfortunately, the pathology data for Mayo clinic cohort were not shared on Synapse. To evaluate our VC-TWAS method performance in the real data, we presented the qq plot for all VC-TWAS results in the application studies, see Fig S6, Fig S10, and Fig S11. These qq plots all show good behavior by VC-TWAS.

The significant associations identified by meta VC-TWAS were mainly driven by the Mayo Clinic Data.

“11 of these genes were shown to possess significant pleiotropic effects on brain pathology phenotypes.”
This significance is based on a p-value threshold of 0.05 (Table 3, Figure 2B) and has not been adjusted for multiple testing of 11 genes and three phenotypes/outcomes.

We want to thank the Reviewer 3 for this suggestion. In this revision, we used Bonferroni correction with respect to 13 genes and 3 phenotypes to claim possible significant pleitropy association (see Table 2).

Minor comments:
Typo: Page 15, first line, CPU *munities*;
Page 19, line 457, “using individual-level DATA for”? “Data” was omitted.

We really appreciate the Reviewer 3 for catching these typos. We have carefully proofread this revision to eliminate grammar errors and typos.
