# Supplementary material for: Novel Variance-Component TWAS method for studying complex human diseases with applications to Alzheimer’s dementia
Source: PLoS Genet. 2021 Apr 2;17(4):e1009482. doi: 10.1371/journal.pgen.1009482 (PMC8046351; doi:10.1371/journal.pgen.1009482)
Supplement: Attachment — Submitted filename: review2.docx [file pgen.1009482.s021.docx]

Thank authors for detailed and comprehensive replies. I still have some comments.

1. In practice, many TWAS methods consider cis-SNPs to be either upstream or downstream 500kb with the range of a gene and the number of cis-SNPs ranges from tens of a few hundreds. In Table S1, the number of cis-SNPs considered ranges from 1000 to >5000. First, authors need to justify that increasing cis-SNP number can enhance power. Otherwise, I don’t see any points to consider such a large number of cis-SNPs to compare the computational efficiency. At least, authors need to show that using such a large of cis-SNPs in VC-TWAS can outperform CoMM/CoMM-S2 that uses a few hundred cis-SNPs. I guess authors are using imputed SNPs in the analysis. If so, authors need to justify that using imputed SNPs can improve power in TWAS over the genotyped SNPs. Second, to further account for horizontal pleiotropy, there are couple of transcriptome-wide Mendelian randomization methods proposed, e.g., PMR-Egger [1] that primary assumes a burden assumption. It will be also interesting to compare performance of VC-TWAS with PMR-Egger as well. In PMR-Egger, the authors compare the computational efficiency in Table 2 [1], but Table S1 is significantly different from that one. This point should be addressed seriously by the authors.

Overall, without justification for a large of cis-SNPs as Table S1, it is pretty aggressive to state as in the paragraph between lines 358 and 365 (as well between lines 109 and 111), which are absolutely misleading for readers as CoMM was designed to consider only a few hundreds genotyped cis-SNPs, especially such conclusions are based on the incorrect Table S1. Moreover, readers need to know whether they should use imputed cis-SNP or genotyped cis-SNP. If the power from genotyped cis-SNP is comparable to that of imputed cis-SNP, there is no reason to make comparisons in Table S1. I suggest the authors revise the parts I mentioned here.

2. According to CoMM, “The analysis for an individual trait in the NFBC1966 dataset can be done around 2 min and around 18 min for an individual disease in the GERA dataset on a Linux platform with 2.6GHz intel Xeon CPU E5-2690 with 30 720 KB cache and 96 GB RAM (only 7.8 GB RAM used) on 24 cores.” In CoMM, the authors assume that the number of cis-SNPs is less than sample size which makes sense as the median of the number of cis-SNPs (500kb on each side) in GWAS is around 200. For this, CoMM solves the problem using the X’X, which X is genotype and the computation is in order less than O(p^3). If the number of cis-SNPs is larger than the sample size, it is optimal to solve using XX’ and the computation is at most O(n^3). This is IMPORTANT based on your assumptions for the number cis-SNPs. In Table S1, we should expect at most 73*5^3 = 9125 seconds for SNP number > 5000 as I explained above. But the reported time is 10 times that of expected. Again, the results in Table S1 here is significantly different from Table 2 reported in PMR-Egger [1]. Thus, it must be somethings wrong here and authors must correct any mistakes for this table. As PMR-Egger is an extension considering a fixed intercept (burden test), authors may compare it as well. For a fair comparison, authors need to state clearly for the assumptions that all methods made. It will be helpful to have a table of summary for pros, cons, and any assumptions among different methods.
3. Though CoMM was developed using linear models, it can still be applied to binary outcome. The differences between using linear and logistic regression would not be that much as long as the case-control ratio is too extreme which is the case in this study. At least, it does not hurt to compare. Moreover, the sentence in introduction between lines 108 and 109 should be rewritten. It is more informative to state the assumption of VC-TWAS as the binary outcome and CoMM as the continuous outcome. It is not necessary that a linear model cannot be applied to the studies with binary outcomes. Please revise the corresponding statement in the manuscript.
4. To promote reproducibility, all codes for results (tables and figures) in this manuscript should be provided, e.g. in github.
5. Provided the parallel implementation, it is computationally feasible using CoMM for large sample size as GERA (PMR-Egger even used UKB data) so it would be interesting to compare CoMM/CoMM-S2 as well as PMR-Egger with VC-TWAS in real data analysis. Even without parallelization, it would at most take 18/60*24=7.2 hours to complete analysis for a trait in GERA that is much larger than the sample used in this manuscript.

[1] Yuan, Z., Zhu, H., Zeng, P., Yang, S., Sun, S., Yang, C., ... & Zhou, X. (2020). Testing and controlling for horizontal pleiotropy with probabilistic Mendelian randomization in transcriptome-wide association studies. *Nature communications*, *11*(1), 1-14.
