# Supplementary material for: Novel Variance-Component TWAS method for studying complex human diseases with applications to Alzheimer’s dementia
Source: PLoS Genet. 2021 Apr 2;17(4):e1009482. doi: 10.1371/journal.pgen.1009482 (PMC8046351; doi:10.1371/journal.pgen.1009482)
Supplement: Attachment — Submitted filename: Response_R2_03_03_2021.docx [file pgen.1009482.s022.docx]

In this response letter, we have carefully addressed Reviewer #2’s comments about justification of using imputed or WGS SNP data within +-1MB test gene region, explaining discrepancy between the computation time table in our manuscript and the ones in PMR-Egger and moPMR-Egger paper, discussing the potential inflation of type I error when applying linear mixed model to dichotomous traits, and providing real data analysis results with COMM and PMR-Egger methods. Please find the point-by-point responses as follows.

Reviewer #2: Thank authors for detailed and comprehensive replies. I still have some comments.
1. In practice, many TWAS methods consider cis-SNPs to be either upstream or downstream 500kb with the range of a gene and the number of cis-SNPs ranges from tens of a few hundreds. In Table S1, the number of cis-SNPs considered ranges from 1000 to >5000. First, authors need to justify that increasing cis-SNP number can enhance power. Otherwise, I don’t see any points to consider such a large number of cis-SNPs to compare the computational efficiency. At least, authors need to show that using such a large of cis-SNPs in VC-TWAS can outperform CoMM/CoMM-S2 that uses a few hundred cis-SNPs. I guess authors are using imputed SNPs in the analysis. If so, authors need to justify that using imputed SNPs can improve power in TWAS over the genotyped SNPs. Second, to further account for horizontal pleiotropy, there are couple of transcriptome-wide Mendelian randomization methods proposed, e.g., PMR-Egger [1] that primary assumes a burden assumption. It will be also interesting to compare performance of VC-TWAS with PMR-Egger as well. In PMR-Egger, the authors compare the computational efficiency in Table 2 [1], but Table S1 is significantly different from that one. This point should be addressed seriously by the authors.
Overall, without justification for a large of cis-SNPs as Table S1, it is pretty aggressive to state as in the paragraph between lines 358 and 365 (as well between lines 109 and 111), which are absolutely misleading for readers as CoMM was designed to consider only a few hundred genotyped cis-SNPs, especially such conclusions are based on the incorrect Table S1. Moreover, readers need to know whether they should use imputed cis-SNP or genotyped cis-SNP. If the power from genotyped cis-SNP is comparable to that of imputed cis-SNP, there is no reason to make comparisons in Table S1. I suggest the authors revise the parts I mentioned here.

**Response:** We thank the reviewer for these comments. We respond to the list of concerns outlined above as follows:

*A) Window Size:* We use a 1 MB window size to define cis-acting SNPs, which adheres to the same guidelines used by the GTEx consortium (Nature 2017: 550: 204). Existing TWAS tools, including the original PrediXcan and FUSION TWAS methods (Gamazon et. al., 2015 Nature Genetics; Gusev et. al. 2016 Nature Genetics) as well as the UTMOST method (Hu et al. 2019 Nature Genetics), likewise implement similar 1-MB window sizes. Consequently, many applied TWAS projects also assume a 1 MB window (e.g. Raj et al. 2018; Nature Genetics 50: 1584; Mancuso et al. Nature Communications 9: 4079). Thus, we think our choice of a 1 MB window size to define cis-SNPs is well justified.

*B) Use of Imputed SNPs in TWAS (with Additional Discussion of Scaling to WGS data):* To show the importance of considering cis-SNPs in a +-1MB region around the test gene and incorporating imputed genotypes, we trained gene expression prediction models by both PrediXcan and TIGAR/DPR methods using only genotyped SNPs in a +-500MB region around the test gene (the CoMM strategy), and genotyped and imputed SNP data in a +-1MB region around the test gene (the VC-TWAS strategy). For each method, we trained the gene expression prediction models using samples from the ROS cohort and then tested using samples from the MAP cohort. The prediction R2 were calculated as the squared correction between the predicted and profiled gene expression. As shown in the following Figure 1, the prediction accuracy of GReX when using genotyped SNPs in a +-500MB region relative to genotyped and imputed SNP data in a +-1MB region varies with respect to the prediction model applied. For PrediXcan prediction models, the GReX prediction accuracy is significantly improved by using imputed SNP data (Figure 1A). For the TIGAR/DPR method, the use of imputed SNPs resulted in similar performance for genes with relatively low prediction R2 (<0.2) but still resulted in slightly improved prediction R2 for genes with relatively high prediction R2 (>0.2) (Figure 1B). Thus, overall, we conclude that leveraging imputed SNPs in a larger window size around the gene of interest does enhance the quality of TWAS.

Figure 1. Comparison of prediction accuracy of genetically regulated gene expression component in real ROS/MAP data by both PrediXcan and TIGAR methods using array genotyped SNP data, imputed SNP data, or WGS SNP data.

Related to the discussion of leveraging imputed SNPs in TWAS, we wish to note that the GTEx reference dataset (which forms the backbone of most TWAS methods and TWAS applied analyses) possesses whole-genome sequencing (WGS) data such that most genes in this dataset will harbor significantly more cis-SNPs than the few hundred currently presumed by CoMM, even if only considering a +-500KB window around the test gene. To show the value of including all SNPs from WGS in a TWAS, we trained similar gene expression prediction models as before using TIGAR/DPR in a +-1MB region around the test gene but used WGS data rather than imputed data for the analysis. Using the WGS SNP data improves on the use of imputed data (Figure 1C). Likewise, we observed improved prediction R2 by using WGS SNP data in +-1MB gene region versus using only genotyped SNP data in a +-500MB region, especially for genes with relatively high prediction R2 (>0.2) (Figure 1D).

We hope the above results emphasize the importance of incorporating large number of cis-acting SNPs into TWAS. We also wish to note that VC-TWAS is scalable to handle even larger gene sets that leverage both cis- and trans-SNPs (such as those identified by BGW-TWAS), which we illustrated in our manuscript using the IGAP dataset. Given interest in exploring trans effects in general, we believe VC-TWAS will have practical value in exploring such hypotheses.

*C) Computational Comparisons with PMR-Egger:* After inspecting the computation times listed in Table 2 of the PMR-Egger paper [1] and Table S4 of the moPMR-Egger paper [2], we would like to point out that they both are not directly comparable with Table S1 in this manuscript. One issue is that PMR-Egger and moPMR-Egger defined cis-SNPs as those SNPs within 100KB around the gene and were further found in both GEUVADIS training data and UK Biobank test data. This definition of cis-SNPs differs substantially from our broader definition that we use in this manuscript, as noted in previous sections A and B of this response. Consequently, the median number of SNPs per gene considered by PMR-Egger/moPMR-Egger is substantially smaller than what we considered in this manuscript. For example, the median number of cis-SNPs considered by PMR-Egger in GEUVADIS that are shared with WTCC, GERA, or UK Biobank is 200, 556, and 500, respectively, while the mean number of cis-SNPs considered by moPMR-Egger that are shared between GEUVADIS and UK Biobank traits is 497 (median = 443, min=1, max = 8085). In contrast, in our VC-TWAS application studies with real data, the median number of cis-SNPs per gene was 2872; a greater than five-fold increase over the median number of SNPs consider by PMR-Egger/moPMR-Egger.

Another issue is that, in Table 2 in PMR-Egger paper, the PrediXcan and TWAS methods compared in the paper conduct Burden TWAS test as described in our manuscript, but the computation time shown in Table 2 of the PMR-Egger paper should include the gene expression model training step. Whereas, in Table S1 of our manuscript, our computation time table only shows the Burden test step and did not account for the computational cost for model training. The Table 2 in PMR-Egger paper does not clearly state the numerical steps that form the computation time unit used in the table.

We did apply PMR-Egger method with our ROS/MAP genotyped SNP data. We found that the computation time of PMR-Egger is similar to the CoMM method, which is consistent as shown in Table 2 in PMR-Egger paper. The discrepancy we see between Table 2 in PMR-Egger paper and our computation time table could be partially due to different CPU specs. We have added our CPU specs to our table legend. We also added an additional row in our Table S1 to show the computation time for all methods when there are only ~100-300 SNPs.

[1] Yuan, Z., Zhu, H., Zeng, P., Yang, S., Sun, S., Yang, C., ... & Zhou, X. (2020). Testing and controlling for horizontal pleiotropy with probabilistic Mendelian randomization in transcriptome-wide association studies. Nature communications, 11(1), 1-14.

[2] Liu L, Zeng P, Xue F, Yuan Z, Zhou X. Multi-trait transcriptome-wide association studies with probabilistic Mendelian randomization. The American Journal of Human Genetics. 2021 Feb 4;108(2):240-56.

2. According to CoMM, “The analysis for an individual trait in the NFBC1966 dataset can be done around 2 min and around 18 min for an individual disease in the GERA dataset on a Linux platform with 2.6GHz intel Xeon CPU E5-2690 with 30 720 KB cache and 96 GB RAM (only 7.8 GB RAM used) on 24 cores.” In CoMM, the authors assume that the number of cis-SNPs is less than sample size which makes sense as the median of the number of cis-SNPs (500kb on each side) in GWAS is around 200. For this, CoMM solves the problem using the X’X, which X is genotype and the computation is in order less than O(p^3). If the number of cis-SNPs is larger than the sample size, it is optimal to solve using XX’ and the computation is at most O(n^3). This is IMPORTANT based on your assumptions for the number cis-SNPs. In Table S1, we should expect at most 73*5^3 = 9125 seconds for SNP number > 5000 as I explained above. But the reported time is 10 times that of expected. Again, the results in Table S1 here is significantly different from Table 2 reported in PMR-Egger [1]. Thus, it must be somethings wrong here and authors must correct any mistakes for this table. As PMR-Egger is an extension considering a fixed intercept (burden test), authors may compare it as well. For a fair comparison, authors need to state clearly for the assumptions that all methods made. It will be helpful to have a table of summary for pros, cons, and any assumptions among different methods.

**Response:** We want to thank the reviewer for describing the computation complexity of the CoMM method. We used the CoMM tool provided in the original paper. We followed all instructions as given by the tool. Our simulation studies showed that CoMM tool was well calibrated and had the best power which ensures that the tool is used correctly.

The reviewer states that computation time for CoMM is expected as most to be 73*5^3=9125 seconds for SNP number > 5000 but it is unclear to us how the reviewer is deriving this quantity. The reviewer never formally defined what the numbers 73 and 5 refer to and it is unclear why the cubic exponent is only applied to the number 5, when (based on the reviewer’s description) it should be applied to the quantity consisting of min(sample size, number of cis-SNPs). We are also not sure if the sample size in the computation complexity O(n^3) is for the training sample size or test sample size.

We would like to point out that the number of cis-SNPs we considered in our data analysis has median ~2K, while our training sample size is 499 and our test sample size is 1287. Our computation time was obtained by a single thread with 4 cores in a 2.10 GHZ 16-core Intel Xeon CPUs with 512 GB RAM. We have clarified these in our table legend. Regarding the differences between the computation time reported in Table 2 in PMR-Egger paper and our Table S1, please see section C of our response to the first comment. Basically, the number of test cis-SNPs is different (with median 200, 556, and 500 in PMR-Egger paper and median 2872 in our real analysis).

In this paper, our motivation is to provide an alternative variance component TWAS test than the existing Burden type TWAS test. Thus, we thoroughly compared our methods with the Burden type TWAS test methods. We have discussed the CoMM/PMR-Egger method in our introduction and discussion sections (lines 108-113 on page 5; lines 591-604 on page 25 with tracking) to point out the shared random effect size assumption by both CoMM and VC-TWAS, the advantage of CoMM/PMR-Egger method by jointly modeling both training and test data, and the computation issue of CoMM/PMR-Egger method for testing thousands of SNPs per gene. We think we have been fair to existing methods. Our motivation is not to beat CoMM method in the power perspective, but to provide an alternative tool that can address the limitation of existing burden test with computation efficiency for handling imputed or WGS GWAS data. We think our main text have well addressed the pros and cons of existing burden methods, as well as CoMM/PMR-Egger.

3. Though CoMM was developed using linear models, it can still be applied to binary outcome. The differences between using linear and logistic regression would not be that much as long as the case-control ratio is too extreme which is the case in this study. At least, it does not hurt to compare. Moreover, the sentence in introduction between lines 108 and 109 should be rewritten. It is more informative to state the assumption of VC-TWAS as the binary outcome and CoMM as the continuous outcome. It is not necessary that a linear model cannot be applied to the studies with binary outcomes. Please revise the corresponding statement in the manuscript.

**Response:** While some GWAS studies have applied linear mixed models (LMMs) to dichotomous outcomes with little issue, the validity of this strategy in general is not guaranteed. As the reviewer noted, application of LMMs to unbalanced case-control data can lead to problematic inference. Also, as noted by Chen et al [3] (AJHG 98: 653), the homoscedasticity assumption required by LMMs is inherently violated for dichotomous outcomes in the common situation when such outcomes depend on covariates (such as ancestry). Using both simulated and real data, Chen et al. showed that the violation of this important homoscedasticity assumption for dichotomous outcomes lead to LMM test statistics that were poorly calibrated. The authors instead show that a logistic mixed model is properly calibrated and they therefore recommend the use of logistic procedures for large-scale GWAS of dichotomous outcomes to ensure validity. We have rewritten lines 108-109 in our introduction (see lines 108-113 on page 5 with tracking) and also added a paragraph in our discussion section (lines 591-604 on page 25 with tracking) clarifying these points.

[3] Chen H, Wang C, Conomos MP, Stilp AM, Li Z, Sofer T, et al. Control for Population Structure and Relatedness for Binary Traits in Genetic Association Studies via Logistic Mixed Models. American journal of human genetics. 2016;98(4):653-66. Epub 2016/03/29. doi: 10.1016/j.ajhg.2016.02.012.

4. To promote reproducibility, all codes for results (tables and figures) in this manuscript should be provided, e.g. in github.

**Response:** All of our source codes will be shared on Github once our paper is accepted for publication. Our TIGAR tool that implements VC-TWAS function has already been made available on Github, <https://github.com/yanglab-emory/TIGAR>.

5. Provided the parallel implementation, it is computationally feasible using CoMM for large sample size as GERA (PMR-Egger even used UKB data) so it would be interesting to compare CoMM/CoMM-S2 as well as PMR-Egger with VC-TWAS in real data analysis. Even without parallelization, it would at most take 18/60*24=7.2 hours to complete analysis for a trait in GERA that is much larger than the sample used in this manuscript.

**Response:** We want to thank the reviewer for this suggestion. We found that the number of test cis-SNPs rather than sample size was the main driver of the computational cost for VC-TWAS as well as for CoMM/PMR-Egger methods. As we mentioned in our response to the first comment, the PMR-Egger only considered SNPs within 100KB around the gene as cis-SNPs that are found in both GEUVADIS training data and UK Biobank test data, such that the median number of cis-SNPs per gene was ~500. For VC-TWAS, we assumed a broader definition for cis-SNPs such that the median number of tested cis-SNPs per gene was 2872 in our application studies.

As the reviewer suggested, we applied both CoMM/CoMM-S2 and PMR-Egger methods to Chromosome 19 in our real data (499 reference samples with individual-level data and IGAP summary statistics), where we used only genotyped SNP data within +-500KB around the test gene (since a +-1MB window was too computationally demanding). Since the median number of tested cis-SNPs is now 147 (see the following Figure 2), both CoMM/CoMM-S2 and PMR-Egger methods do finish within hours.

As shown in the following Figure 3, all three methods identified similar number of significant genes that are near the major APOE locus and majority of these significant genes are identified by more than one method, e.g., 31 out of 44 significant genes by CoMM-S2 are also identified by VC-TWAS, and 23 genes are identified by all three methods. As shown in the Figure 3 in the main text, those nearby significant genes tend to have highly correlated GReX components and might show the same biological mechanism underlying the APOE locus.

As shown in our response for the first comment, we found that GReX prediction accuracy on imputed data is similar to the accuracy using genotype data alone when using TIGAR/DPR but that leveraging WGS SNP data should improve accuracy. In this paper, we only used imputed genotype data. Thus, we would expect the TWAS results by VC-TWAS and CoMM method would be similar. Again, our goal in this paper is to provide an alternative tool to relax the limitation by the existing Burden type TWAS methods with efficient computation. We feel a comparison with CoMM method using only genotyped data in real analysis is beyond the scope of this paper.

Figure 2. Number of tested cis-SNPs using only genotyped SNP data on Chromosome 19.

Figure 3. Manhattan plot for the TWAS results by VC-TWAS, CoMM, PMR-Egger methods, using ROS/MAP training data and the IGAP AD GWAS summary statistics.

Table 1:

Table 2:
