# Supplementary material for: Novel Variance-Component TWAS method for studying complex human diseases with applications to Alzheimer’s dementia
Source: PLoS Genet. 2021 Apr 2;17(4):e1009482. doi: 10.1371/journal.pgen.1009482 (PMC8046351; doi:10.1371/journal.pgen.1009482)
Supplement: Attachment — Submitted filename: review3.docx [file pgen.1009482.s023.docx]

Thank authors for detailed and comprehensive numerical studies for this revision. I still have some minor comments for tones of writing.

1. In the paragraph starting on line 363, authors should change the word “600x” to “up to 600x” and also need to mention that when summary-level data is used, the fold change is only “up to 250x”.
2. It will be helpful to provide a summary of related methods. For example, a table summarizes all the pros and cons of related methods.
