# Supplementary material for: Novel Variance-Component TWAS method for studying complex human diseases with applications to Alzheimer’s dementia
Source: PLoS Genet. 2021 Apr 2;17(4):e1009482. doi: 10.1371/journal.pgen.1009482 (PMC8046351; doi:10.1371/journal.pgen.1009482)
Supplement: Attachment — Submitted filename: Response_R3_03_11_2021.docx [file pgen.1009482.s024.docx]

We thank Reviewers and Editors for considering our last revision is sufficient to address all reviewers’ comments. In this minor revision, we changed our description of “existing TWAS methods” to “traditional two-stage TWAS methods”, in order to be more accurately referring only to those Burden-TWAS methods.

Reviewer #2: Thank authors for detailed and comprehensive numerical studies for this revision. I still have some minor comments for tones of writing.

1. In the paragraph starting on line 363, authors should change the word “600x” to “up to 600x” and also need to mention that when summary-level data is used, the fold change is only “up to 250x”.

We thank the Reivewer2 for suggesting more precisely to describe the computation cost comparison between VC-TWAS and CoMM methods. We have edited our descriptions as suggested.

2. It will be helpful to provide a summary of related methods. For example, a table summarizes all the pros and cons of related methods.

We thank the Reivewer2 and Editors for suggesting this summary table. We have added such a summary table in Table S5 and referred to in our Discussion section.
